# Identification of TPRA1 as a Novel Receptor and Predictive Biomarker for Oncolytic Virus M1

**DOI:** 10.1002/advs.202510597

**Published:** 2025-10-06

**Authors:** Linyi Hu, Guigen Zhang, Yuan Lin, Shiming Yi, Jingyu Yang, Xueying Lin, Deli Song, Zhiheng Liu, Jiayu Zhang, Ying Zeng, Shanyu Huang, Zhen Fan, Jifu Zhang, Ying Liu, Cheng Hu, Wenbo Zhu, Wei Yin, Jun Hu, Guangmei Yan, Jing Cai, Jiankai Liang

**Affiliations:** ^1^ Department of Biochemistry Zhongshan School of Medicine Sun Yat‐sen University Guangzhou 510080 China; ^2^ Department of Microbiology Zhongshan School of Medicine Sun Yat‐sen University Guangzhou 510080 China; ^3^ Guangmei Yan, Department of Pharmacology Zhongshan School of Medicine Sun Yat‐sen University Guangzhou 510080 China; ^4^ School of Life Sciences Peking University Beijing 100871 China; ^5^ The Sixth Affiliated Hospital of Sun Yat‐sen University Guangzhou 510655 China; ^6^ The Third Affiliated Hospital of Sun Yat‐sen University Guangzhou 510630 China

**Keywords:** biomarker, oncolytic virus M1, receptor, TPRA1, viral entry

## Abstract

Viral receptors are essential host factors that determine the tropism of oncolytic viruses, and contribute to their selective targeting of cancer cells. In this study, a membrane protein‐targeted CRISPR‐Cas9 screen is conducted and identify transmembrane protein adipocyte‐associated 1 (TPRA1) as a novel receptor for oncolytic virus M1(OVM), a promising oncolytic virus currently under clinical investigation. Mechanistically, TPRA1 facilitates OVM infection by promoting both viral attachment and internalization. Extracellular region of TPRA1 directly binds OVM particles via glycosylation, while its cytoplasmic tail mediates virus endocytosis, collectively enabling efficient viral entry and cancer cell lysis. Importantly, TPRA1 expression in cell lines, mouse models, and patient‐derived tumor samples are positively correlated with their respective sensitivity to OVM, and TPRA1 is upregulated in a high proportion of tumors compared to adjacent normal tissues, highlighting its potential as a therapeutic response biomarker. Furthermore, TPRA1 also promotes the entry of Semliki Forest Virus, suggesting its conserved role in alphavirus infection. Together, these findings establish TPRA1 as both a mechanistic determinant of OVM tropism and a biomarker to guide patient selection in clinical trials of OVM‐based therapy.

## Graphical abstract

This study identifies transmembrane protein adipocyte‐associated 1 (TPRA1) as a novel receptor for oncolytic virus M1 (OVM). TPRA1's ectodomain binds OVM particles, while its intracellular domain facilitates virus internalization, promoting efficient viral entry. TPRA1 expression positively correlates with OVM therapeutic efficacy, highlighting TPRA1 as a predictive biomarker for precision OVM‐based therapy.

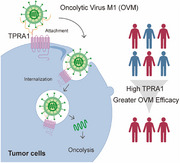

## 1 Introduction

Oncolytic viruses (OVs) are a class of naturally occurring or engineered viruses that selectively target and destroy tumor cells while sparing normal tissue.^[^ ^1^ ^]^ By replicating within tumor cells, OVs induce immunogenic cell death and stimulate antitumor immunity, demonstrating significant therapeutic potential.^[^ ^2^, ^3^, ^4^, ^5^ ^]^ More than 10 virus families have been explored for use as OVs, with several advancing through clinical trials for cancer treatment.^[^ ^6^ ^]^ Key milestones include the approval of the HSV‐1 derived oncolytic virus Talimogene laherparepvec (T‐VEC)^[^ ^7^ ^]^ in 2015 and Delytact (teserpaturev) in 2021.^[^ ^8^ ^]^ Despite these achievements, clinical outcomes remain highly variable, largely due to the genetic heterogeneity of tumors.^[^ ^9^ ^]^ This underscores the critical need for further exploration of OV‐tumor interactions and the identification of key host factors to establish precise and effective oncolytic virus therapies.

Viral receptors are among the most critical host factors that determine the cell tropism of viruses, shaping their ability to infect specific cell types. For example, angiotensin‐converting enzyme 2 (ACE2) serves as the receptor for SARS‐CoV‐2. High expression of ACE2 in specific epithelial cells of lungs, heart, kidneys, and intestines provides SARS‐CoV‐2 with strong tissue tropism for these organs.^[^ ^10^ ^]^ Similarly, chemokine receptor 5 (CCR5) is the primary co‐receptor used by human immunodeficiency virus (HIV) to enter macrophages and CD4^+^ T cells during the early stages of infection.^[^ ^11^ ^]^ Individuals carrying the CCR5 gene mutation (CCR5Δ32) are less susceptible to infection, offering protection against AIDS.^[^ ^12^ ^]^ In the context of oncolytic viruses, the expression of these receptors on tumor cells is important for achieving tumor selectivity. The presence, abundance, and accessibility of viral receptors significantly influence the efficiency of OV entry and subsequent virus replication and oncolysis within tumor cells. T‐VEC, an HSV‐1‐based oncolytic therapy, has demonstrated efficacy in melanoma,^[^ ^7^ ^]^ partially attributable to the elevated expression of its cognate receptor Nectin‐1 on melanoma cells.^[^ ^13^ ^]^

OVM, a strain of Getah‐like alphavirus, is a naturally occurring oncolytic virus with an 11.6 kb positive‐sense single‐stranded RNA genome encoding four nonstructural proteins (NSP1, NSP2, NSP3, NSP4) and five structural proteins (C, E3, E2, 6K, E1).^[^ ^14^, ^15^ ^]^ Preclinical studies have shown that OVM directly lyses tumor cells while simultaneously activating a potent anti‐tumor immune response, demonstrating strong efficacy across various solid tumor models.^[^ ^16^, ^17^, ^18^, ^19^, ^20^ ^]^ Furthermore, OVM can be administered intravenously, enabling systemic delivery and precise targeting of metastatic tumors.^[^ ^21^ ^]^ These distinct advantages have advanced OVM therapy to Phase I clinical trials in China (NCT06368921) and Japan (NCT06046742), underscoring its potential as a safe and effective oncolytic virotherapy.

OVM also exhibits strong tropism for tumor cells while sparing normal cells, a property partially attributed to factors such as the deficiency of the antiviral protein ZAP or the presence of KRAS mutations, both of which enable efficient virus replication in tumor cells.^[^ ^14^, ^22^ ^]^ MXRA8 has been identified as a receptor for OVM, and the combination of MXRA8 expression and ZAP deficiency in cancer cells significantly enhances the virus's anti‐tumor efficacy.^[^ ^23^ ^]^ However, we observed that some cells lacking MXRA8 expression can still be infected by OVM, indicating the involvement of additional receptors.

In this study, we employed a membrane protein‐targeted CRISPR‐Cas9 screen to identify transmembrane protein adipocyte‐associated 1 (TPRA1) as a critical factor for OVM infection. Our findings demonstrate that TPRA1 interacts with OVM particles via its ectodomain, while its intracellular C‐terminal domain facilitates OVM internalization, thereby enhancing viral entry into tumor cells. We establish TPRA1 as a novel receptor for OVM, significantly boosting its anti‐tumor efficacy both in vitro and in vivo. These results highlight TPRA1 as a potential biomarker for predicting the therapeutic efficacy of OVM.

## 2 Results

### 2.1 CRISPR‐Cas9 Screening Identifies TPRA1 as a Critical Host Factor for OVM Infection

MXRA8 has been identified as a receptor for OVM,^[^ ^23^ ^]^ however, we found that some cell lines, such as HEK293, which do not express MXRA8, remained susceptible to OVM infection (Figure S1A, Supporting Information). Similarly, human breast cancer Hs 578T cells retained sensitivity to OVM infection even when MXRA8 is knocked out (Figure S1B,C, Supporting Information). These findings strongly suggest the existence of additional, unidentified receptors that mediate OVM entry.

**Figure 1:**
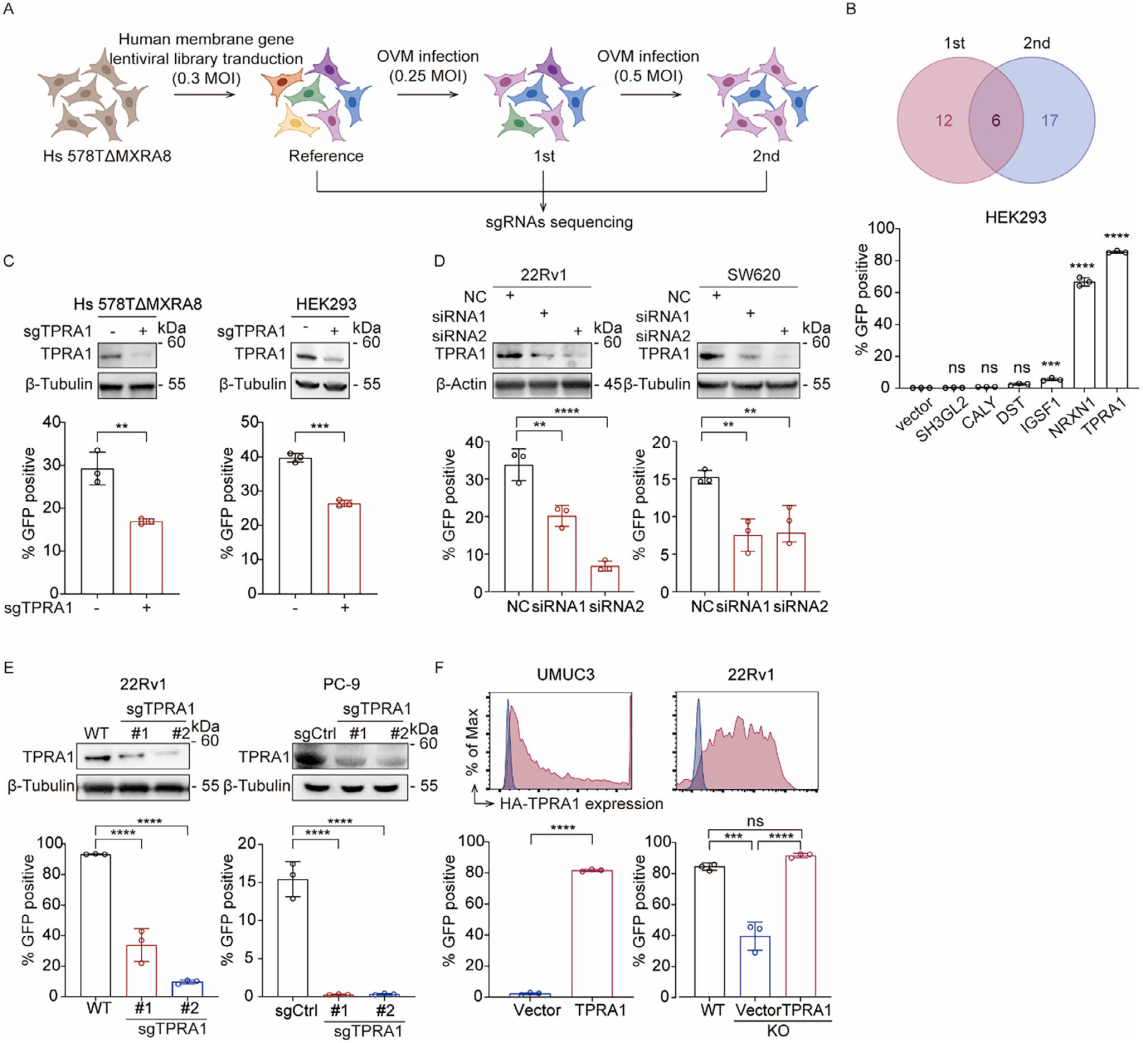
CRISPR‐Cas9 Screens Identify TPRA1 as a Crucial Host Factor for OVM Infection. A) Hs 578TΔMXRA8 cells transduced with a membrane protein‐targeting sgRNA library underwent two rounds of OVM selection (1st and 2nd). Surviving cells were sequenced and compared to the unchallenged library (Reference) to identify enriched sgRNAs. B) Six candidate genes enriched in both rounds of the OVM challenges were overexpressed in HEK293 cells, which were infected by OVM expressing GFP reporter (OVM‐GFP, 0.01 MOI) and quantified for GFP‐positive cells at 48 h post‐infection (hpi) by flow cytometry. C) TPRA1 knockout in Hs 578TΔMXRA8 and HEK293 cells reduced OVM‐GFP infection (1 MOI, 24 hpi for Hs 578TΔMXRA8; 2 MOI, 24 hpi for HEK293). D) TPRA1 siRNA knockdown in 22Rv1/SW620 cells decreased infection (1 MOI, 48 hpi). E) TPRA1 KO in 22Rv1/PC‐9 cells reduced OVM‐GFP infection (1 MOI, 48 hpi for 22Rv1; 1 MOI, 24 hpi for PC‐9). F) HA‐TPRA1 expression in 22Rv1 KO/UMUC3 cells restored infection (0.1 MOI, 48 hpi for UMUC3; 1 MOI, 48 hpi for 22Rv1). HA expression was confirmed by flow cytometry. The data are representative of three experiments. *P* values were determined by Student's *t*‐test or one‐way ANOVA. ns, not significant; ^*^ *P* < 0.05; ^**^ *P* < 0.01; ^***^ *P* < 0.001; and ^****^ *P* < 0.0001.

To identify an alternative receptor, we performed CRISPR‐Cas9 loss‐of‐function screens in MXRA8 knock‐out Hs 578T cells (Hs 578TΔMXRA8). Lentiviruses delivering single guide RNAs (sgRNAs) targeting human membrane protein‐encoding genes^[^ ^24^ ^]^ were used to transduce Hs 578TΔMXRA8 cells to construct library cells (**Figure** **1**A). The library cells were subjected to two rounds of OVM selection, and cells that survived each round were collected for genomic DNA extraction. The sgRNA‐coding regions were amplified, deep‐sequenced, and analyzed to compare their abundance to that in uninfected library cells (Figure S2A,B, Supporting Information). We focused on genes with sgRNAs enriched in both rounds of OVM selection, and identified six candidates: CALY, SH3GL2, DST, IGSF1, NRXN1, and TPRA1. By overexpressing these genes in HEK293 cells, we found that TPRA1 most potently enhances OVM infection (Figure 1B). TPRA1 (also termed FLJ32197, GPR175, TMEM227, and TPRA40) is an orphan G protein‐coupled receptor (GPCR) reported to play roles in embryonic cleavage and the negative regulation of mitotic cell cycle phase transition,^[^ ^25^, ^26^, ^27^, ^28^ ^]^ but its potential role in viral‐host interactions remains unexplored. Consistent with the screen results, CRISPR‐Cas9‐mediated disruption of TPRA1 in Hs 578TΔMXRA8 and HEK293 cells also significantly reduced OVM infection (Figure 1C and Figure S3A, Supporting Information).

To confirm the role of TPRA1 in OVM infection, we demonstrated that silencing TPRA1 in prostate cancer 22Rv1 cells and colon cancer SW620 cells using siRNA significantly reduced virus infection rate (Figure 1D). Similarly, CRISPR‐Cas9‐mediated disruption of TPRA1 in 22Rv1 and lung cancer PC‐9 cells resulted in a substantial decrease in OVM infection (Figure 1E). Conversely, ectopic expression of TPRA1 in bladder cancer UMUC3 cells, which showed less TPRA1 expression, rendered them more susceptible to OVM infection, and trans‐complementation of TPRA1 in 22Rv1ΔTPRA1 cells fully restored OVM infectivity (Figure 1F and Figure S3B, Supporting Information). Collectively, these findings establish TPRA1 as a critical host factor for OVM infection.

### 2.2 TPRA1 Enhances OVM Attachment and Internalization

**Figure 2:**
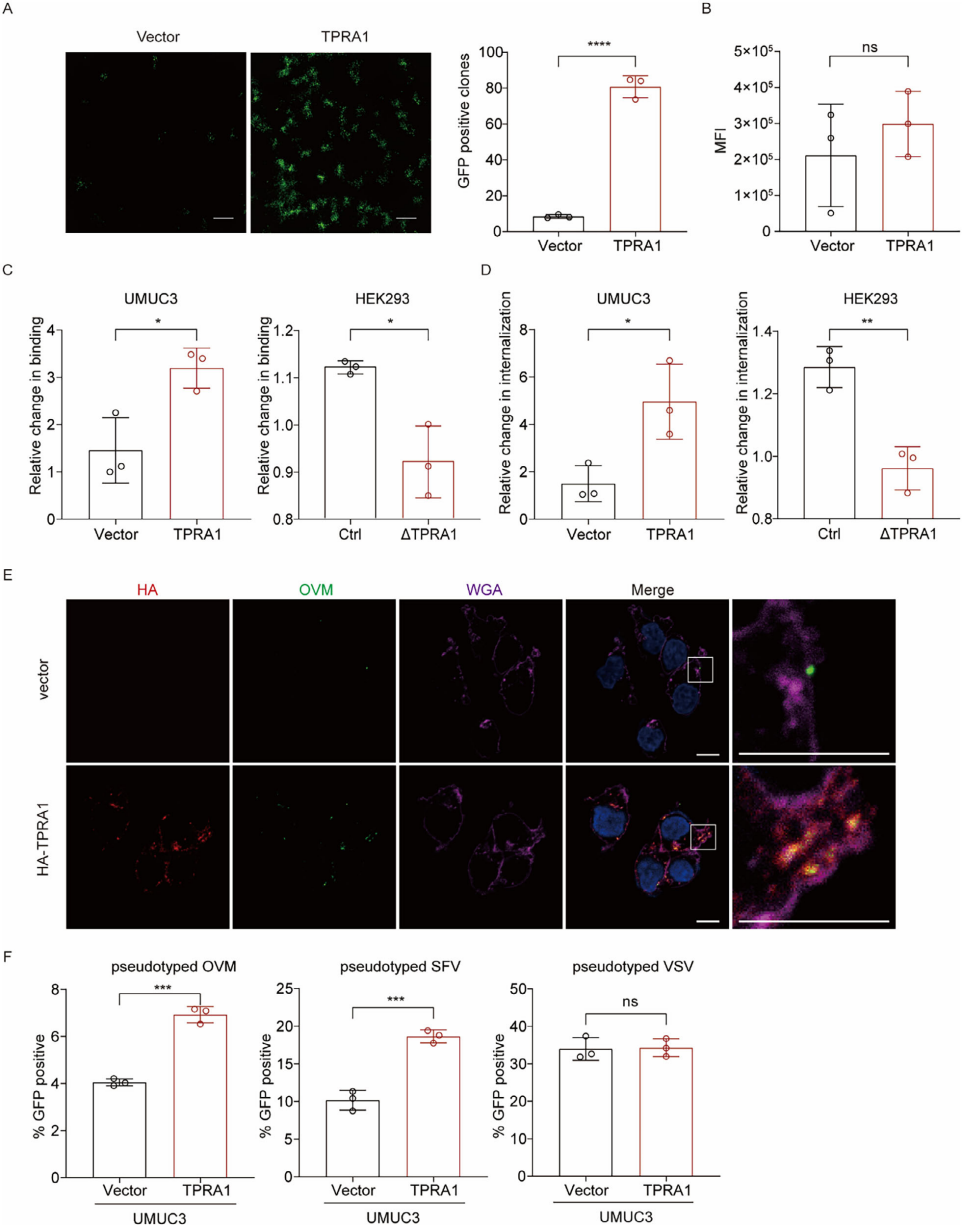
TPRA1 Promotes OVM Attachment and Internalization. A) UMUC3 cells overexpressing TPRA1 or vector were treated with OVM‐GFP for 1 h, followed by replacement with semisolid medium. GFP‐positive clones were scanned at 48 hpi. Scale bar: 500 µm. B) OVM RNA was transfected into control or TPRA1‐overexpressing UMUC3 cells. Cells were analyzed for GFP reporter expression at 48 h post‐transfection. C,D) UMUC3 cells overexpressing TPRA1 or vector, and HEK293ΔTPRA1 or control cells were incubated with OVM at 4 °C for 1 h (C) or shifted to 37 °C for 1 h (D). Cells were then collected, and OVM RNA was quantified by qRT‐PCR using β‐Actin as a control. E) Biotin‐labeled OVM were incubated with TPRA1‐overexpressing or vector UMUC3 cells at 4 °C for 1 h and 37 °C for 5 min, followed by immunofluorescence analysis. Scale bar: 10 µm. F) Lentiviral particles (encoding a GFP reporter) pseudotyped with structural proteins from OVM, SFV, or VSV were used to infect UMUC3 cells overexpressing TPRA1 or vector control cells. GFP‐positive cells, indicative of successful viral entry, were quantified by flow cytometry. The data are representative of three experiments. *P* values were determined by Student's *t*‐test. ns, not significant; ^*^ *P* < 0.05; ^**^ *P* < 0.01; ^***^ *P* < 0.001; and ^****^ *P* < 0.0001.

To investigate the mechanism by which TPRA1 enhances OVM infection, we first conducted a plaque formation‐based entry assay, and significantly more plaques were observed in TPRA1‐overexpressing UMUC3 cells (**Figure** **2**A). However, when OVM genomic RNA was directly transfected into control and TPRA1‐overexpressing UMUC3 cells in the presence of NH_4_Cl, which inhibits virus maturation and subsequent rounds of infection, no differences in reporter gene expression were observed (Figure 2B). These results suggest that TPRA1 promotes the viral entry stage rather than the replication stage of OVM. To confirm this, OVM binding at 4 °C was obviously increased in TPRA1‐overexpressing UMUC3 and 22Rv1 cells compared to control cells (Figure 2C and Figure S4A, Supporting Information). Similarly, Virus internalization assays performed at 37 °C revealed significantly enhanced OVM uptake in TPRA1‐overexpressing UMUC3 and 22Rv1 cells (Figure 2D and Figure S4B, Supporting Information). In contrast, TPRA1 knock‐out in HEK293 cells resulted in reduced OVM binding and internalization (Figure 2C,D), further supporting this observation. Direct visualization using biotin‐labeled OVM revealed that more OVM particles were taken up by TPRA1‐overexpressing cells, with OVM consistently colocalizing with TPRA1 (Figure 2E). Furthermore, the infection of OVM pseudotyped virions encapsulating a lentivirus GFP‐reporter RNA was also significantly enhanced in TPRA1‐overexpressing UMUC3 cells, with a similar pattern observed for pseudotyped virions of Semliki Forest Virus (SFV), an old world alphavirus closely related to OVM. In contrast, pseudotyped virions of Vesicular Stomatitis Virus (VSV), a negative‐sense RNA virus belonging to the vesiculovirus genus, did not show enhanced infection in TPRA1‐overexpressing cells (Figure 2F). Together, these findings demonstrate that TPRA1 promotes OVM entry by increasing virus attachment and internalization.

### 2.3 TPRA1 Binds to OVM Directly

**Figure 3:**
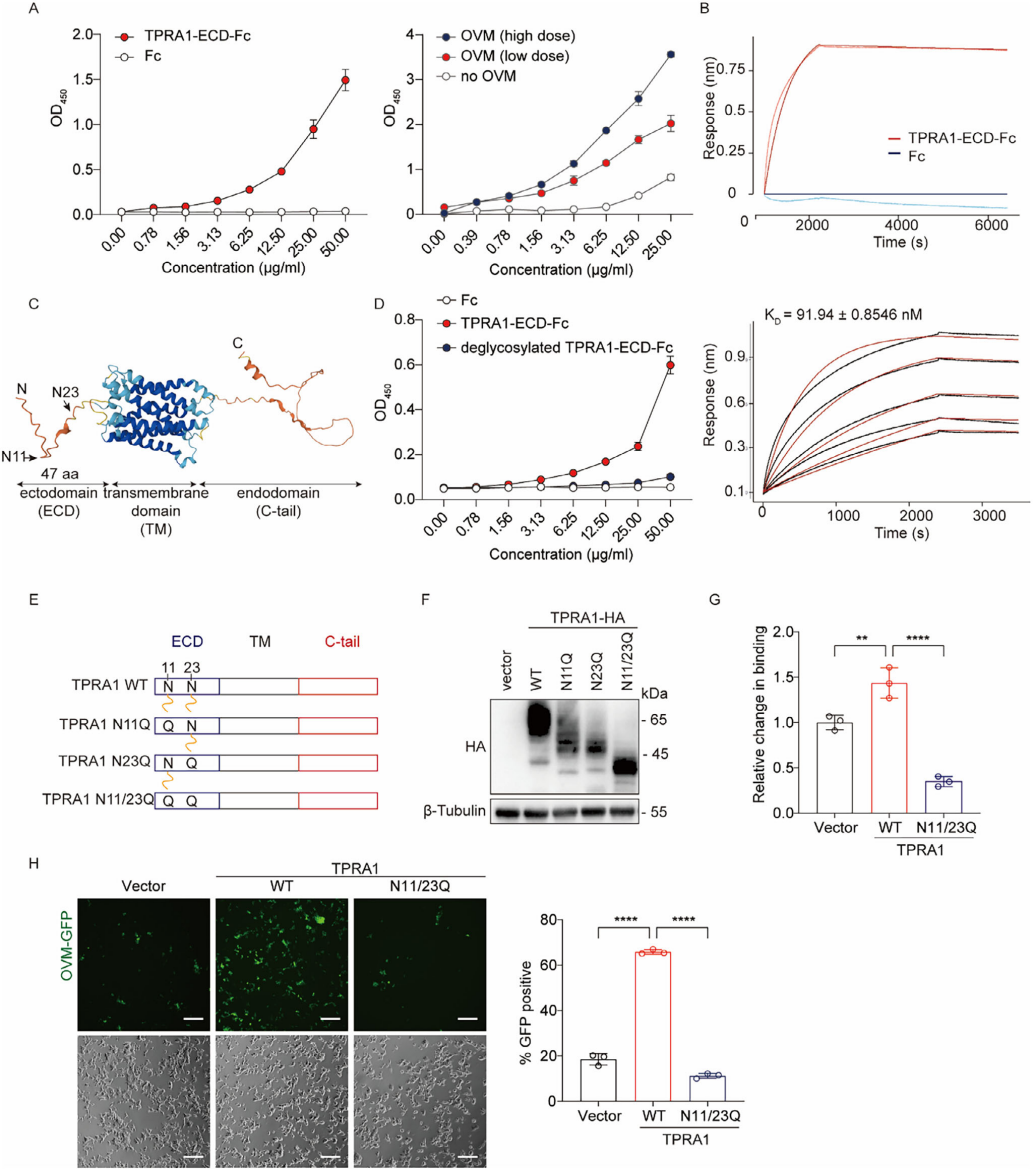
TPRA1 Binds Directly to OVM. A) Left, serially diluted TPRA1‐ECD‐Fc or control Fc proteins were added to an ELISA plate coated with OVM to assess binding. Right, serially diluted TPRA1‐ECD‐Fc proteins were added to ELISA plates coated with high‐dose (5×10^6^ TCID50), low‐dose (2×10^6^ TCID50), or no OVM to assess binding. B) Upper, BLI sensorgrams of TPRA1‐ECD‐Fc or Fc proteins (8 µm) binding to OVM‐coated sensor tips. Lower, sensorgrams depicting TPRA1‐ECD‐Fc (0.5, 1, 2, 4, or 8 µm) binding to OVM‐coated tips. C) Predicted TPRA1 structure by Alphafold. D) Fc, TPRA1‐ECD‐Fc, or PNGase F‐treated TPRA1‐ECD‐Fc (deglycosylated) were added to OVM‐coated ELISA plates to assess binding. E) Schematic of TPRA1 mutants with N11 or N23 in the ectodomain mutated to Q. F) Immunoblot of HA‐tagged TPRA1 variants in 22Rv1 cells. Images are representative of two independent experiments. G) OVM binding of 22Rv1 cells expressing TPRA1 variants, measured by qRT‐PCR. H) 22Rv1 cells expressing TPRA1 variants were infected with OVM‐GFP (0.1 MOI) for 48 h. Left, microscopy images showing infected cells (GFP‐positive). Right, quantification of GFP‐positive cells by flow cytometry. The data are representative of three experiments. *P* values were determined by one‐way ANOVA. ^**^ *P* < 0.01; and ^****^ *P* < 0.0001.

To determine whether TPRA1 directly interacts with OVM, we performed an ELISA‐based binding assay using the recombinant extracellular domain of TPRA1 fused to an Fc tag (TPRA1‐ECD‐Fc). The results showed that TPRA1‐ECD‐Fc bound to OVM virions in a dose‐dependent manner, whereas Fc alone exhibited no binding (**Figure** **3**A). This interaction was further confirmed by Biolayer Interferometry (BLI), where TPRA1‐ECD‐Fc displayed an affinity of ≈91 nm, while Fc alone did not bind to OVM (Figure 3B).

TPRA1 exhibits the canonical seven transmembrane domains of a GPCR, with a short N‐terminal extracellular region (47 amino acids) and a C‐terminal intracellular domain (87 amino acids) (Figure 3C). Given the limited size of its extracellular region, we hypothesized that post‐translational modifications might enhance TPRA1's binding to the virus. Sequence analysis of TPRA1 (UniProtKB: Q86W33) predicted only two post‐translational modifications within this region: N‐glycosylation sites at asparagine (N)11 and N23. Therefore, we hypothesized that the N‐linked glycans in TPRA1's extracellular region might contribute to OVM binding. To assess the role of glycosylation in OVM binding, we treated TPRA1‐ECD‐Fc with PNGase F, an enzyme that removes N‐linked oligosaccharides, and found that this treatment completely abolished OVM binding, suggesting that N‐linked glycans are critical for OVM binding (Figure 3D).

To confirm the role of N‐linked glycosylation, we generated TPRA1 mutants in which N11 or N23 were substituted with glutamine (Q) (Figure 3E). Immunoblot analysis revealed increased electrophoretic mobility of the mutants, consistent with the loss of glycosylation at these sites and confirming the presence of these modifications (Figure 3F). Cells expressing TPRA1 with both mutations (N11/23Q) showed significantly reduced OVM binding and lower infection rates compared to those expressing wild‐type TPRA1 (Figure 3G,H).

To examine whether OVM E1/E2 directly recognize N‐linked glycans, we performed glycan array screening, which contained 100 glycans enriched for GlcNAc residues—the foundational sugar of N‐glycosylation (Table S1, Supporting Information). Purified OVM particles bound to a subset of glycans, with stronger signals for intact virions than for E1 or E2 proteins alone (Figure S5A, Supporting Information), suggesting that the assembled viral spike structure optimizes glycan engagement. Notably, OVM showed the strongest binding to glycan G0015 [Gal‐β‐1,3‐(Fuc‐α‐1,4)‐GlcNAc‐β, Lewis A] (Figure S5B, Supporting Information), a terminal sequence on N‐glycan chains. Lewis antigens are frequently overexpressed on cancer cells, where they contribute to tumor progression, metastasis, and therapy resistance.^[^ ^29^ ^]^ They are also well‐established pathogen attachment factors, recognized by viruses such as noroviruses.^[^ ^30^ ^]^ Moreover, OVM, as well as E1 and E2, also bound to glycan G0014 [Neu5Gc‐α‐2,6‐Gal‐β‐1,3‐GlcNAc‐β] (Figure S5B, Supporting Information). Neu5Gc‐containing glycoconjugates are observed in many tumors and contribute to tumor development and progression.^[^ ^31^ ^]^

These findings demonstrate that TPRA1 facilitates OVM infection by directly binding to the virus, and this interaction is critically dependent on the N11 and N23 glycosylation sites in the extracellular region of TPRA1.

### 2.4 TPRA1 Endocytosis Facilitates OVM Internalization and Infection

**Figure 4:**
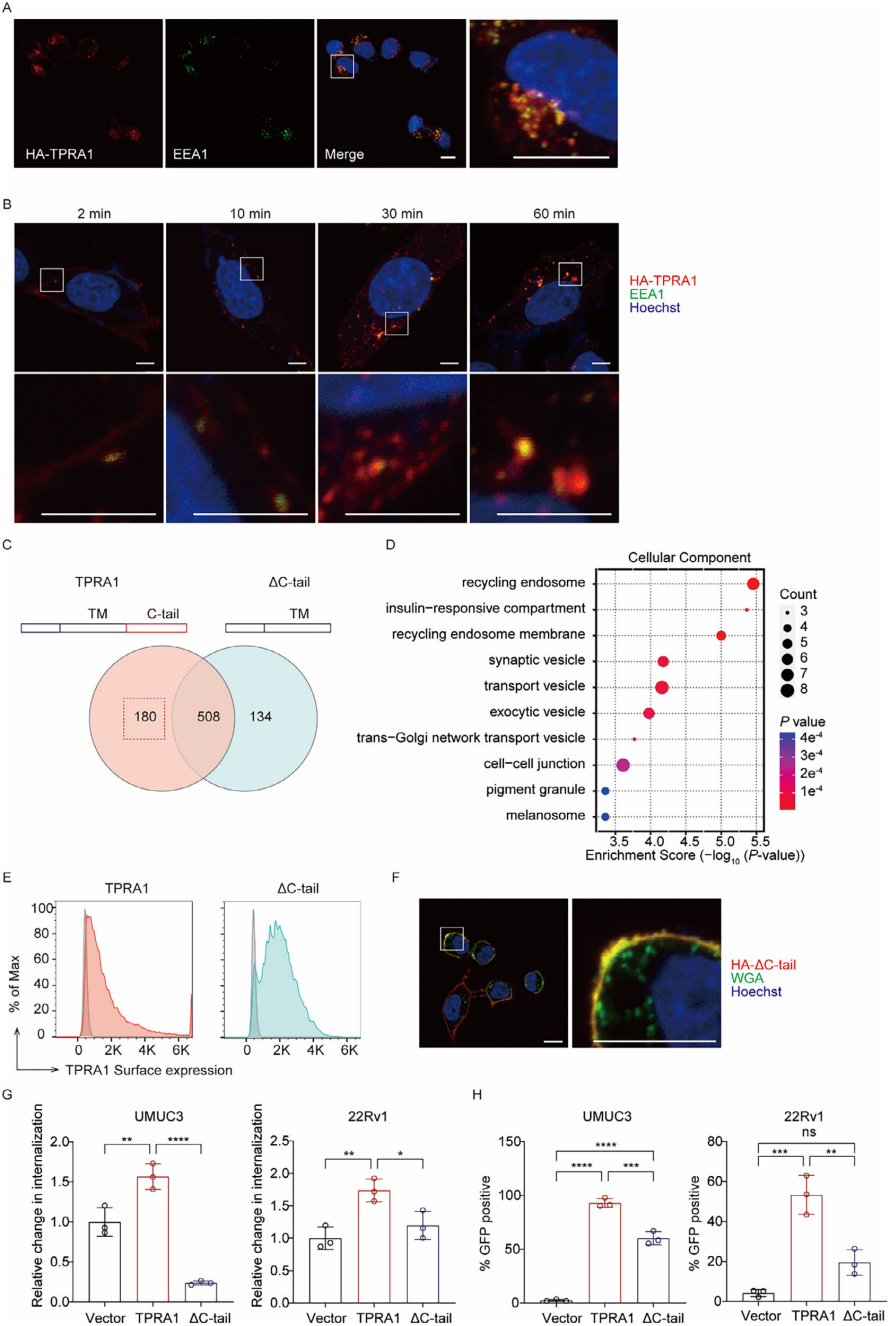
TPRA1 Endocytosis Is Necessary for Promoting OVM Infection. A) Confocal immunofluorescence microscopy of ectopically expressed HA‐TPRA1 and early endosome marker EEA1 in UMUC3 cells. Scale bar: 10 µm. B) Cells were incubated with anti‐HA antibodies at 4 °C for 1 h, then warmed to 37 °C. Cells were fixed at respective time points, and anti‐HA antibodies were visualized to track TPRA1 trafficking. Scale bar: 5 µm. C) Co‐IP/mass spectrometry analysis of HA‐tagged full‐length or ΔC‐tail TPRA1‐interacting proteins in UMUC3 cells. Left circle: 688 proteins interacting with the full‐length TPRA1; right circle: 642 proteins interacting with the ΔC‐tail TPRA1; square: 180 proteins specifically interacting with the full‐length TPRA1 but not the ΔC‐tail. D) Gene ontology analysis of 180 C‐tail interacting proteins. E) Flow cytometry detecting surface HA‐TPRA1 or HA‐ΔC‐tail in UMUC3 cells. F) Confocal microscopy of HA‐ΔC‐tail in UMUC3 cells. WGA: Wheat germ agglutinin; scale bar: 10 µm. G) OVM internalization of UMUC3 or 22Rv1 cells expressing vector, TPRA1, or ΔC‐tail analyzed by qRT‐PCR. H) UMUC3 or 22Rv1 cells stably expressing vector, TPRA1 or ΔC‐tail, were infected with OVM‐GFP (0.1 MOI) for 48 h. The data are representative of three experiments. *P* values were determined by one‐way ANOVA. ns, not significant; ^*^ *P* < 0.05; ^**^ *P* < 0.01; ^***^ *P* < 0.001; and ^****^ *P* < 0.0001.

Information from the Human Protein Atlas^[^ ^32^ ^]^ indicates that TPRA1 is primarily localized in vesicles. Immunofluorescence staining revealed that TPRA1 was present on both the cell surface and within intracellular compartments, with a strong association with the early endosome marker EEA1 (**Figures** **4**A and S6A, Supporting Information). In contrast, TPRA1 did not colocalize with the late endosome marker Rab7 or the lysosome marker LAMP1 (Figure S6A,B, Supporting Information).

GPCRs are known to undergo internalization and recycling. Upon activation by a ligand—such as a neurotransmitter, chemokine, or pathogen like a virus—GPCRs recruit intracellular effectors, including GPCR kinases (GRKs) and β‐arrestins, through their intracellular loops. This process facilitates the subsequent recruitment of endocytic proteins, such as adaptor protein 2 (AP2) and clathrin, leading to clathrin‐mediated endocytosis and GPCR recycling.^[^ ^33^, ^34^ ^]^ Influenza A, Ebola, and Marburg Virus have been shown to exploit GPCRs to facilitate viral entry.^[^ ^35^, ^36^ ^]^ In immunofluorescence assays, we also observed the colocalization of TPRA1 and OVM (Figure 2E). Thus, we hypothesize that TPRA1, as a GPCR, may be such a recycling receptor that OVM exploits for internalization. To investigate whether TPRA1 functions as a recycling receptor, we first tracked its trafficking by labeling the cell surface pool of HA‐tagged TPRA1 with anti‐HA antibodies under cold conditions. Cells were warmed to initiate endocytosis and fixed at defined time points. Labeled TPRA1 progressively translocated from the plasma membrane to intracellular compartments, colocalizing with EEA1 (Figure 4B and Figure S7A, Supporting Information). Moreover, we observed that internalized TPRA1 can return to the plasma membrane (Figure S7B,C, Supporting Information), indicating its recycling from the endosome to the cell surface. Interestingly, the intracellular trafficking route of TPRA1 closely mirrored that of alphaviruses, both moving from the plasma membrane to the endosome.^[^ ^37^, ^38^ ^]^ Next, we investigated whether TPRA1 translocates from the plasma membrane to the early endosome via motifs in its C‐terminal cytoplasmic tail (C‐tail), as observed in many other proteins.^[^ ^39^ ^]^ We expressed HA‐tagged full‐length TPRA1 or a C‐tail deletion mutant (ΔC‐tail) in UMUC3 cells and analyzed C‐tail interacting proteins via co‐immunoprecipitation (co‐IP) and mass spectrometry (Figure 4C). Gene Ontology (GO) analysis showed that these C‐tail‐interacting proteins are enriched in recycling endosomes and transport vesicles (Figure 4D). Consistently, deletion of the C‐tail resulted in increased TPRA1 surface expression (Figure 4E and Figure S7D, Supporting Information) and impaired its endocytosis (Figure 4F and Figure S7E, Supporting Information). Notably, ΔC‐tail expressed in cells exhibited a deficiency in mediating OVM internalization and infection compared to wild‐type TPRA1(Figure 4G,H). These data demonstrate that the C‐terminal cytoplasmic tail of TPRA1 mediates its cycling between the plasma membrane and endosome, thereby facilitating OVM internalization.

To further explore the molecular mechanism of TPRA1‐mediated OVM entry, we used Arbidol, a clathrin‐dependent endocytosis inhibitor, and found that Arbidol can partially inhibit the infection of OVM, suggesting TPRA1‐mediated entry via the clathrin pathway (Figure S8, Supporting Information). Furthermore, based on candidate interactors identified in our co‐IP/MS experiments, we excluded nonspecific binders and performed siRNA screens targeting 99 genes (Figure S9A, Supporting Information) to identify key functional players. In two independent rounds of screening, we identified 19 genes whose knockdown consistently reduced OVM‐induced cell death by more than 20% in both experiments (Figure S9B, Supporting Information). Functional annotation revealed that four of these genes (TUBB4B, RAB13, RAB8A, and ARFGAP1) are closely related to endosome formation and trafficking, supporting their potential role in OVM internalization. Further validation confirmed that knockdown of these candidates significantly reduced OVM internalization (Figure S9C,D, Supporting Information), which consequently led to a substantial decrease in OVM infection, with knockdown of TUBB4B exhibiting the most potent effect (Figure S9E,F, Supporting Information). TUBB4B, a major constituent of microtubules that serve as tracks for vesicle and organelle transport, has been reported to be involved in both viral and bacterial entry and internalization.^[^ ^40^, ^41^ ^]^ RAB13 and RAB8A, as small Rab GTPases, are key regulators of intracellular membrane traffickingt.^[^ ^42^ ^]^ RAB13 primarily functions in transport vesicles formation and fusion and has been reported to participate in the entry step of hepatitis c virus infection.^[^ ^43^ ^]^ RAB8A mediates polarized vesicular trafficking and has been implicated in the entry of dengue virus.^[^ ^44^, ^45^ ^]^ ARFGAP1, a component of the COPI complex, has also been shown to promote AP2‐dependent endocytosis.^[^ ^46^ ^]^ Collectively, our data suggest that the TPRA1 C‐tail interacts with a core set of endocytic machinery to facilitate viral entry.

### 2.5 TPRA1 Enhances the Antitumor Efficacy of OVM

**Figure 5:**
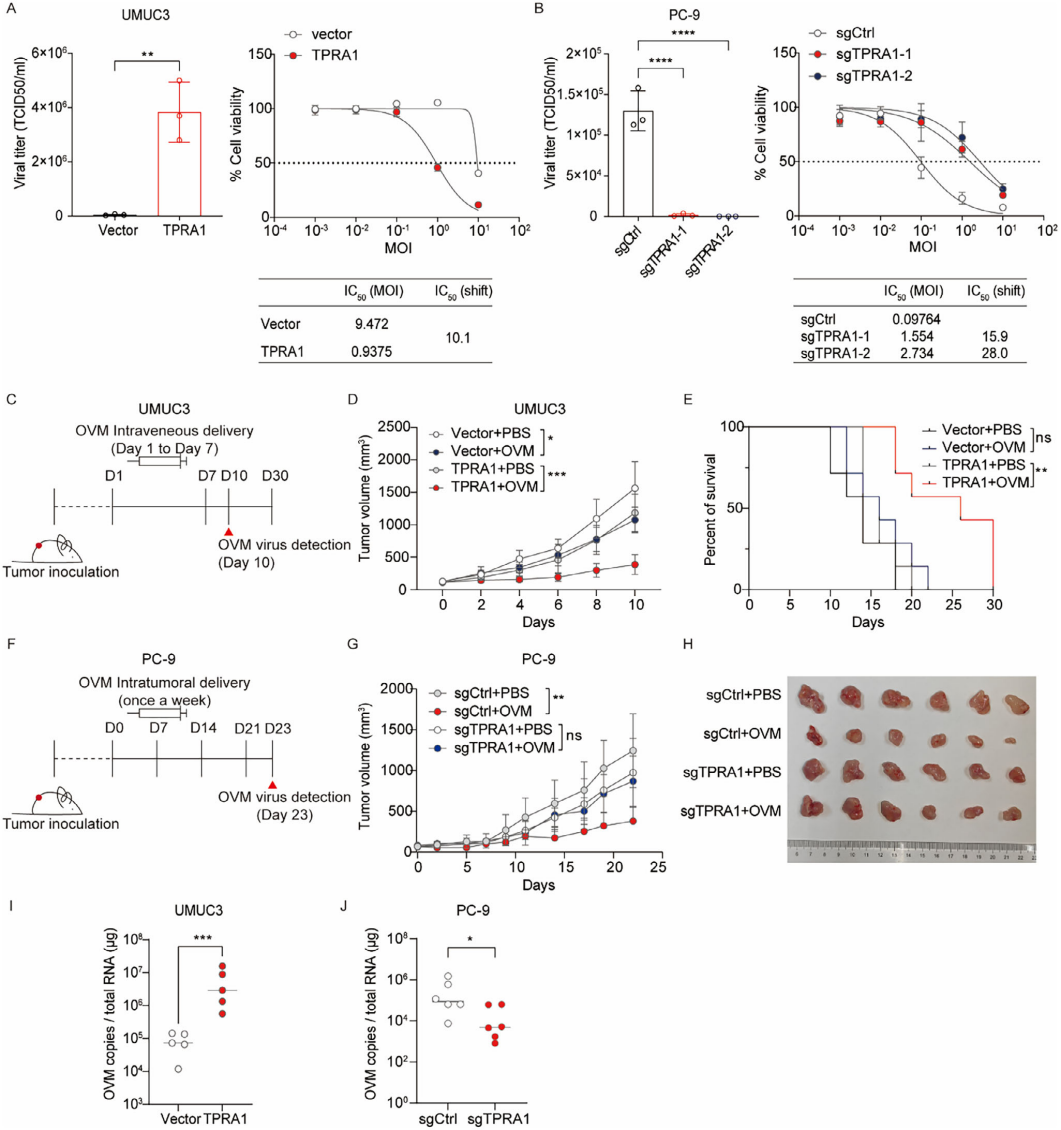
TPRA1 Enhances the Antitumor Efficacy of OVM. A,B) Left: viral titer in supernatant of UMUC3 cells expressing vector or TPRA1 A) and control or ΔTPRA1 PC‐9 cells B) treated with OVM (0.1 MOI) for 72 h. Right: cells were treated with OVM at the indicated MOI, and cell viability was assessed 72 hpi. Data are representative of three independent experiments. C) UMUC3 cells stably expressing TPRA1 or vector control were implanted subcutaneously into BALB/c nude mice. n = 7 mice. Mice received daily intravenous injections of OVM or PBS control for 7 days. OVM abundance in the tumor was detected at day 10. D) Tumor growth curves of UMUC3 tumors. E) Kaplan‐Meier survival curves for UMUC3 tumor‐bearing mice by the log‐rank test. F) Control or ΔTPRA1 PC‐9 cells were implanted subcutaneously into BALB/c nude mice. n = 6 mice. Mice received intratumoral injections of OVM or PBS control once a week. OVM abundance in the tumor was detected at day 23. G) Tumor growth curves of PC‐9 tumors. H) Photograph of PC‐9 tumor tissues at day 23. D: day. I) OVM abundance in UMUC3 tumors overexpressing TPRA1 or vector was detected by qRT‐PCR. J) OVM abundance in ΔTPRA1 or control PC‐9 tumor tissues detected by qRT‐PCR. *P* values were determined by Student's *t*‐test, one‐way ANOVA, or log‐rank test. ns, not significant; ^*^ *P* < 0.05; ^**^ *P* < 0.01 and ^***^ *P* < 0.001.

To assess whether TPRA1‐mediated enhancement of OVM infection results in increased viral replication and oncolytic activity, we first measured virus titers and cell viability in vitro. Ectopic expression of TPRA1 in UMUC3 cells significantly enhanced OVM replication and subsequent cancer cell death (**Figure** **5**A). Conversely, TPRA1 knockout in PC‐9 cells resulted in reduced viral replication and diminished OVM‐induced cytotoxicity (Figure 5B). To further confirm the role of TPRA1 in OVM's antitumor potential in vivo, we found that OVM treatment led to stronger antitumor effects in TPRA1‐overexpressing UMUC3 tumors and prolonged survival (Figure 5C–E). In contrast, in the PC‐9 tumor model, the therapeutic efficacy of OVM was abolished upon TPRA1 knockout (Figure 5F–H). Consistently, UMUC3 tumors overexpressing TPRA1 exhibited significantly higher OVM abundance in the tumor tissue, whereas PC‐9 tumors with TPRA1 knockout showed reduced OVM abundance compared to vector controls (Figure 5I,J). These results demonstrate that TPRA1 expression in tumor cells enhances OVM's therapeutic efficacy both in vitro and in vivo.

### 2.6 TPRA1 Serves as a Biomarker for OVM Therapy

**Figure 6:**
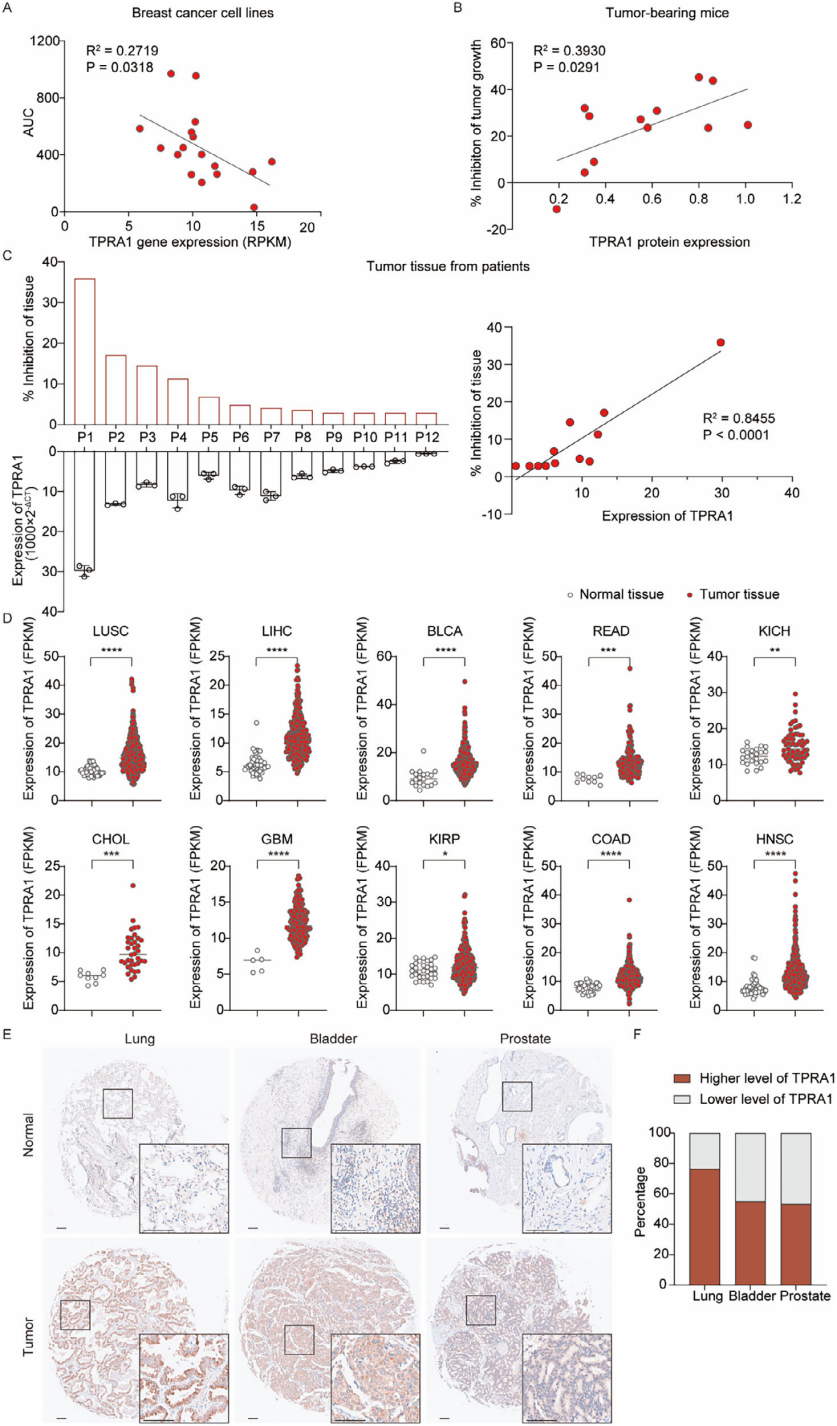
TPRA1 is a Biomarker for Predicting OVM Efficacy. A) OVM anti‐tumor efficacy across breast cancer cell lines correlates with TPRA1 mRNA levels (CCLE). Sensitivity was quantified by the area under the curve (AUC) of cell viability post‐OVM treatment (0, 0.1, 1, 10 MOI). B) TPRA1 protein levels correlate with OVM‐induced growth inhibition in 12 syngeneic mouse tumors (A20, CT26, EMT6, H22, Renca, B16BL6, B16F10, Hepa1‐6, LLC, MC38, Pan02, and RM1). Mice received intravenous OVM or solvent control (200 µL 1×10^7^ TCID50/mL) for 5 days. C) Left, TPRA1 expression and ex vivo OVM efficacy in patient‐derived tumors. TPRA1 expression was measured by qRT‐PCR. P, patient. Right, correlation analysis of TPRA1 expression and ex vivo OVM response. R^2^: Pearson's coefficient of determination. D) TCGA pan‐analysis of TPRA1 mRNA expression levels in tumor versus normal tissues. FPKM: Fragments Per Kilobase of transcript per Million mapped reads. E) Representative TPRA1 immunohistochemistry staining in TMA. Scale bar: 100 µm. F) Percentage of higher TPRA1 or lower TPRA1 in tumors compared with paired normal tissues in three kinds of tumors. Lung, n = 68; Bladder, n = 29; Prostate, n = 45. *P* values were determined by Student's *t*‐test. ^*^ *P* < 0.05; ^**^ *P* < 0.01; ^***^ *P* < 0.001; and ^****^ *P* < 0.0001.

As demonstrated above, TPRA1 functions as a viral receptor and enhances OVM‐mediated oncolysis. We hypothesize that TPRA1 expression correlates with OVM therapeutic efficacy, serving as a potential biomarker for predicting treatment response. To test this hypothesis, we first analyzed previously published data on the oncolytic effects of OVM in breast cancer cell lines,^[^ ^47^ ^]^ using the area under the curve (AUC) as a metric to quantify therapeutic efficacy. TPRA1 expression data for these cell lines were obtained from the CCLE database^[^ ^48^ ^]^ to assess the correlation between TPRA1 expression levels and OVM oncolytic effect. A significant negative correlation was observed between TPRA1 expression and AUC values for OVM treatment, indicating that higher TPRA1 expression is associated with greater OVM oncolytic efficacy (**Figure** **6**A). Additionally, in multiple solid tumor‐bearing mouse models, TPRA1 protein expression in tumors positively correlates with the OVM‐induced tumor growth suppression (Figure 6B and Figure S10A, Supporting Information). Furthermore, in a clinical setting, we performed ex vivo experiments on patient‐derived bladder and prostate cancer tissues. OVM's oncolytic efficacy correlated strongly with TPRA1 expression levels in tumor tissues (Figure 6C), further confirming TPRA1 as a predictive biomarker for OVM therapeutic response.

A pan‐cancer analysis of RNA sequencing data from The Cancer Genome Atlas (TCGA)^[^ ^49^ ^]^ and Genotype Tissue Expression Project (GTEx)^[^ ^50^ ^]^ revealed that TPRA1 is upregulated in various tumor types compared to normal tissues, including lung squamous cell carcinoma (LUSC), liver hepatocellular carcinoma (LIHC), bladder urothelial carcinoma (BLCA), rectum adenocarcinoma (READ), kidney chromophobe (KICH), cholangiocarcinoma (CHOL), glioblastoma multiforme (GBM), kidney renal papillary cell carcinoma (KIRP), colon adenocarcinoma (COAD), head and neck squamous cell carcinoma (HNSC) and Pancreatic adenocarcinoma (PAAD) (Figure 6D and Figure S10B, Supporting Information). Consistent with these findings, immunohistochemistry analysis on tissue microarrays of tumor and adjacent normal tissues further demonstrated that TPRA1 expression is selectively upregulated in 76% of lung adenocarcinoma, 55% of bladder urothelial carcinoma, and 53% of prostate adenocarcinoma cases compared to their corresponding normal tissue (Figure 6E,F). A large proportion of tumor tissues showed high levels of TPRA1, suggesting that OVM could be an effective therapeutic agent for these patients. Using TPRA1 as a biomarker to identify these patients could enhance the precision and efficacy of OVM‐based treatments.

## 3 Discussion

In this study, we identify TPRA1 as a novel receptor for OVM, essential for mediating viral infection and amplifying its antitumor efficacy. Mechanistically, the N‐glycosylated ectodomain of TPRA1 directly binds to OVM particles, while its intracellular C‐tail orchestrates endocytic trafficking to the early endosome, collectively driving viral entry into tumor cells. This dual functionality enables efficient OVM replication and subsequent oncolysis. Importantly, TPRA1 expression correlates positively with OVM sensitivity across preclinical models and patient‐derived tumor samples, underscoring its clinical relevance. These findings establish TPRA1 not only as a critical factor for OVM infection but also as a predictive biomarker for patient stratification in OVM‐based therapies.

As shown, TPRA1 is capable of rapid endocytosis from the plasma membrane and traffics to the early endosome, and this process is also hijacked by OVM to facilitate viral internalization through endocytosis. This mechanism is reminiscent of other viral receptors, such as the adeno‐associated virus (AAV) receptor (AAVR),^[^ ^51^ ^]^ which traffics from the plasma membrane to the trans‐Golgi network, a process essential for AAV infection. Similarly, the low‐density lipoprotein receptor (LDLR), a well‐known recycling receptor conserved in evolution, mediates the uptake of specific ligands, including cholesterol and lipoproteins, through receptor‐mediated endocytosis. LDLR is exploited by a variety of viruses, such as vesicular stomatitis virus,^[^ ^52^ ^]^ hepatitis C virus,^[^ ^53^ ^]^ Crimean‐Congo hemorrhagic fever virus,^[^ ^54^ ^]^ and multiple alphaviruses^[^ ^55^ ^]^ to gain viral entry. These highlight the critical role of endocytic receptors in viral entry, as they couple virus binding with endocytosis, enabling efficient infection. Therefore, these receptors may represent evolutionarily conserved “hotspots” for pathogen interaction. Endocytic receptor TPRA1, as an evolutionarily conserved protein,^[^ ^27^ ^]^ represents a favorable target for viruses, potentially explaining its role in OVM entry.

Glycans are commonly exploited as attachment factors by viruses to facilitate their entry into host cells. A classic example is the use of sialic acids, which typically terminate the glycan chains of glycoproteins and glycolipids, as receptors for the influenza A virus.^[^ ^30^ ^]^ Members of the alphavirus genus have been historically reported to utilize proteinaceous receptors or the glycosaminoglycan heparan sulfate as initial attachment factors.^[^ ^56^, ^57^, ^58^ ^]^ However, the direct recognition of specific terminal structures on N‐linked glycans had not been established for alphavirus. Here, we unveil a glycan‐dependent entry mechanism for OVM by demonstrating that the N‐glycosylated ectodomain of TPRA1 is essential for virus binding. Lewis antigen, a well‐defined terminal motif on N‐glycans^[^ ^29^ ^]^ may mediate this process. This finding is particularly intriguing as the members of Lewis antigen are known receptors for other pathogens, including noroviruses.^[^ ^30^, ^59^ ^]^ Our results establish that OVM recognition of TPRA1's N‐glycans is a critical step in viral entry. Furthermore, given that Lewis and related glycan antigens are frequently overexpressed on cancer cell surfaces,^[^ ^29^ ^]^ this newly identified tropism provides a mechanistic rationale for the tumor selectivity of OVM.

Host factors mediating viral entry are pivotal determinants of viral tropism. OVM, a mosquito‐transmitted alphavirus with no reported human pathogenicity, shares structural homology with other alphaviruses.^[^ ^23^, ^60^ ^]^ While several alphavirus receptors have been identified — such as VLDLR and ApoER2 for Semliki Forest virus (SFV), Eastern equine encephalitis virus (EEEV), and Sindbis virus;^[^ ^61^ ^]^ LDLRAD3 for Venezuelan equine encephalitis virus (VEEV);^[^ ^62^ ^]^ NRAMP2 for Sindbis virus;^[^ ^63^ ^]^ and LDLR for multiple alphaviruses^[^ ^55^, ^58^ ^]^—additional receptors likely remain undiscovered. Here, we demonstrate that TPRA1 promotes the entry of SFV pseudotyped virions (Figure 2F), suggesting it may serve as a shared receptor across alphaviruses. Notably, SFV belongs to the old‐world alphaviruses, a group that also includes clinically significant pathogens such as Ross River virus (RRV) and Chikungunya virus (CHIKV). This raises the possibility that TPRA1 may contribute to the infectivity of these pathogenic viruses. Further studies are needed to define TPRA1 as the receptor for other alphaviruses and its potential as a therapeutic target. Given the evolutionary conservation of TPRA1 and its capacity to mediate viral entry, it may also explain the broad host tropism observed in certain alphaviruses.

OVM has shown a favorable efficacy in preclinical models and phase I clinical trials, yet further optimization is needed to harness its full therapeutic potential, which requires deeper mechanistic insights into its tropism and efficacy. Previous studies demonstrate that viral receptor MXRA8 promotes OVM replication and tumor killing, but MXRA8 alone is not sufficient to predict therapeutic outcomes.^[^ ^23^ ^]^ Here, we show that TPRA1 expression robustly correlates with OVM efficacy across in vitro, in vivo, and ex vivo models, serving as an independent biomarker of therapeutic response. The predictive efficiency of TPRA1 likely stems from its ability to autonomously mediate OVM entry by binding virions via its glycosylated ectodomain and driving internalization through its C‐tail. Our identification of TPRA1 as a novel receptor for OVM not only expands the understanding of OVM tropism but also opens new avenues for precision‐guided therapy. Analyses of tumor tissue microarrays and TCGA data reveal frequent TPRA1 upregulation in malignancies, suggesting broad applicability for OVM in diverse cancers. By integrating TPRA1 expression profiling into clinical workflows, clinicians could screen out the potential responsive patients and establish personalized oncolytic virotherapy. Notably, TPRA1 overexpression in tumors contrasts with its low expression in normal tissues, aligning with OVM's oncolytic specificity and favorable safety profile.

TPRA1 is a member of the G protein‐coupled receptor (GPCR) family, a class of proteins with well‐documented roles in cancer biology,^[^ ^64^ ^]^ including tumor growth, metastasis, immune evasion, and therapy resistance. Microarray and TCGA analyses reveal that TPRA1 is upregulated in multiple malignancies, especially in lung tumors (Figure 6E,F), implicating its involvement in oncogenesis. Intriguingly, TPRA1 is different from conventional GPCR classification, as it does not fit into any of the four major subclasses (A, B, C or F).^[^ ^65^ ^]^ Instead, it shares functional parallels with Smoothened (SMO),^[^ ^27^ ^]^ a non‐classical GPCR protein that positively modulates Hedgehog signaling and drives cancers, such as basal cell carcinoma and medulloblastoma when mutated.^[^ ^66^, ^67^, ^68^ ^]^ Like other GPCRs overexpressed in tumors, such as CXCR4 in multiple cancers,^[^ ^69^ ^]^ CCR5 in breast cancer^[^ ^70^ ^]^ and Lysophosphatidic acid receptors in breast and ovarian,^[^ ^71^ ^]^ TPRA1 may also influence oncogenic pathways or tumor microenvironment dynamically. Notably, TPRA1 is an unfavorable prognostic marker in colon adenocarcinoma and liver hepatocellular carcinoma,^[^ ^72^ ^]^ raising the possibility that its upregulation contributes to malignant transformation. Further experiments are needed to confirm its role in tumorigenesis. Whether TPRA1 upregulation drives oncogenesis or is a bystander effect of tumorigenic signaling remains to be determined.

## 4 Conclusion

In summary, we identify TPRA1 as a novel receptor for OVM, mediating viral infection and amplifying its oncolytic efficacy. Beyond its mechanistic role in viral entry, TPRA1 emerges as a robust predictive biomarker for OVM‐based therapies. By bridging fundamental virology and translational oncology, we offer a mechanistic basis for OVM's tropism while unlocking strategies to optimize its clinical application.

## 5 Experimental Section

### Cells and Viruses

HEK293 (CVCL_0045), UMUC3 (CVCL_1783) and 22Rv‐1 (CVCL_1045) cells were obtained from the American Type Culture Collection (ATCC, USA); PC‐9 (CVCL_B260) cells were from Cellcook Biotech (Guangzhou, China); Hs 578T (CVCL_0332) cells were from Jennio Biotech (Guangzhou, China); SW620 (CVCL_0547) cells were from Tongpai Biotech (Shanghai, China). Vero (CVCL_0059), BHK‐21 (CVCL_1914), B16BL6 (CVCL_0157), B16F10 (CVCL_0159), Hepa1‐6 (CVCL_0327), LLC (CVCL_4358), MC38 (CVCL_B288), Pan02 (CVCL_D627), RM1(CVCL_B459), A20 (CVCL_1940), CT26 (CVCL_7254), EMT6 (CVCL_1924), H22 (CVCL_H613) and Renca (CVCL_2174) were kindly provided by Virotech Pharmaceutical Technology Co., Ltd. (Guangzhou, China). All cell lines were authenticated by STR profiling and tested negative for mycoplasma contamination. HEK293, SW620, B16BL6, B16F10, Hepa1‐6, LLC, MC38, Pan02, and RM1 cells were cultured in Dulbecco's Modified Eagle Medium (DMEM) supplemented with 10% fetal bovine serum (FBS). Vero, BHK‐21, and UMUC3 cells were cultured in Minimum Essential Medium (MEM) supplemented with 10% FBS. Additionally, Hs 578T, 22Rv1, PC‐9, A20, CT26, EMT6, H22, and Renca cells were maintained in RPMI‐1640 medium supplemented with 10% FBS. All cell lines were incubated at 37 °C in a humidified atmosphere containing 5% CO_2_. Alphavirus OVM was provided by Guangzhou Virotech Pharmaceutical Technology Co., Ltd. The virus propagated in Vero cells. Briefly, Vero cells (ATCC) were seeded in T75 culture flakes, cultured in MEM medium with 10% FBS. Prior to infection, the medium was replaced with VP‐SFM (Gibco #11 681 020) supplemented with 1% (v/v) Non‐Essential Amino Acids Solution (Gibco #11 140 050) and 2% (v/v) GlutaMAX Supplement (Gibco #35 050 079). When the cells reached ≈80% confluency, they were infected with OVM at a dose of 100 TCID50. The OVM‐treated Vero cells were incubated for 48 or 72 h until a significant amount of cell cytopathic effect was observed. The culture supernatant was collected, and cellular debris was removed by centrifugation at 5000×g. The supernatant was then filtered through a 0.22 µm filter to obtain the OVM stock, which was stored at −80 °C. Viral titers were determined using TCID50 assay in BHK‐21 cells.

### Pooled sgRNA Screen

A focused CRISPR knockout pooled library, comprising 30848 distinct sgRNAs targeting 3283 genes encoding human membrane proteins, was prepared as previously described.^[^ ^24^ ^]^ The sgRNA plasmid library was packaged into lentiviral particles in HEK293T cells by co‐transfecting with pMD2.G (expressing vesicular stomatitis virus glycoprotein, VSV‐G) and pCMVR8.74 (a lentiviral packaging plasmid) using X‐tremeGENE HP DNA transfection reagent (Roche). Viral supernatants were collected, filtered through a 0.22 µm syringe filter, and used for subsequent experiments. The single‐clone Hs 578TΔMXRA8 cell line was generated using a lentiviral vector, as previously described.^[^ ^23^ ^]^ Cas9 nuclease cleavage activity in this cell line was confirmed by T7E1 assays. Hs 578TΔMXRA8 cells were transduced with the lentiviral sgRNA library at a multiplicity of infection (MOI) of 0.3, followed by puromycin selection for 1 week. Approximately 2 × 10^7^ cells were inoculated with OVM at an MOI of 0.25. To block secondary infection by progeny viruses, the medium was replaced with fresh medium containing 10 µg mL^−1^ heparin sodium (Sangon Biotech #A603251) every two days from 24 h post‐infection (hpi) onward. By day 6, most cells had died due to OVM‐induced cytopathic effects. Surviving cells were expanded in fresh medium. Once the population reached ≈10⁷ cells, half were used for genomic DNA extraction, and the other half were re‐infected with OVM at an MOI of 0.5. This procedure was repeated for a second round of OVM selection (0.5 MOI). Genomic DNA was extracted from uninfected control cells (≈1.5 × 10^7^) and surviving cells (≈4 × 10^6^) from the first and second rounds of OVM selection. The sgRNA‐coding regions in the genomic DNA were amplified by PCR (using CaslibF: TATCTTGTGGAAAGGACGAAACACC and CaslibR: AATACGGTTATCCACGCGGC). Amplified products were subjected to deep‐sequencing analysis. The sgRNAs with read counts below 50 in the control library were excluded. Genes with sgRNA read counts enriched more than 15‐fold in the selection process were prioritized for validation.

### Gene Validation

Lentiviral vectors encoding candidate genes were purchased from GeneCopoeia. Lentiviral particles were produced with a standard lentiviral packaging system. Briefly, HEK293T cells were co‐transfected with the lentiviral vector plasmids, pMD2.G, and pCMVR8.74. At 48 h post‐transfection, viral supernatants were collected and filtered through a 0.22 µm syringe filter. The filtered supernatants, containing lentiviral particles, were used to generate stable HEK293 cell lines overexpressing the candidate genes through lentiviral transduction followed by puromycin (0.5 µg mL^−1^) selection. For OVM infection rate evaluations, OVM expressing a GFP reporter (OVM‐GFP) was generated as previously described.^[^ ^37^ ^]^ Cells were inoculated with the OVM‐GFP viruses and harvested using trypsin at the indicated time points. GFP positive cells were quantified by flow cytometry (Beckman Coulter). TPRA1 was further validated sgRNA/Cas9‐mediated gene editing. For HEK293 cells, a clonal HEK293‐Cas9 stable line was generated through lentiviral transduction followed by blasticidin selection. HEK293‐Cas9 and Hs 578TΔMXRA8 cells were transiently transfected with a plasmid expressing sgRNA targeting TPRA1 (TGTAGAAGGTGATAAAAATG). After puromycin selection for seven days, single‐cell clones were seeded into 96‐well plates. For 22Rv1 cells, sgRNAs targeting TPRA1 (CCAGCGCCACCACAAACACC and TGTAGAAGGTGATAAAAATG) were cloned into the PX458 vector (Addgene #48 138) and transiently transfected into 22Rv1 cells. At 48 h post‐transfection, single cells were sorted into 96‐well plates using flow cytometry. For PC‐9 cells, two TPRA1‐targeting sgRNAs (TGTAGAAGGTGATAAAAATG and GAGGUCCCAGUACCGGACCC) and one control sgRNA (ACGGAGGCTAAGCGTCGCAA) were cloned into the eSpCas9‐LentiCRISPR v2 vector (GenScript). Lentiviral particles were produced using a standard lentiviral packaging system. Following lentiviral transduction and puromycin selection for seven days, single cells were sorted into a 96‐well plate using flow cytometry. Clonal cell lines were expanded and screened for TPRA1 expression by Western blot. Cell clones lacking TPRA1 expression were selected for further experiments.

### RNA Interference

Specific and negative control siRNAs were purchased from Ribobio (Guangzhou, China). Transfection of siRNAs was performed using Lipofectamine RNAiMAX (Thermo Fisher#13778‐150) with OPTI‐MEM medium (Thermo Fisher#31 985 070) according to the manufacturer's instructions. The siRNA sequences targeting TPRA1 were as follows: siRNA‐TPRA1‐1: GAACGCTGCAACTGTTGCT; siRNA‐TPRA1‐2: ATCCTCTTCTCCTACAAAT.

### qRT‐PCR

Total RNA from cells or tissues was extracted using TRIzol reagent (Life Technologies) according to the manufacturer's instructions. A total of 2 µg of RNA was reverse transcribed into cDNA using oligo(dT) primers and RevertAid Reverse Transcriptase (Thermo Scientific#EP0442) following the supplier's protocol. Relative quantitative PCR (qPCR) was performed using SuperReal PreMix SYBR Green (TIANGEN#FP205). The relative cDNA levels were calculated using the comparative CT (cycle threshold) method. The primer sequences used were as follows: β‐Actin‐F: GATCATTGCTCCTCCTGAGC; β‐Actin‐R: ACTCCTGCTTGCTGATCCAC; TPRA1‐F: GATCATTGCTCCTCCTGAGC; TPRA1‐R: CTCTGTCTCGTCCACTTGGCAT; M1‐NS1‐F: GTTCCAACAGGCGTCACCATC; M1‐NS1‐R: ACACATTCTTGTCTAGCACAGTCC

### Western Blotting

Cells were collected, lysed, and proteins were extracted. The protein samples were then subjected to electrophoresis on 10% SDS‐PAGE gels. Proteins were transferred to PVDF membranes using the eBlot L1 system (GenScript). The membranes were blocked with 5% non‐fat milk for 2 h at room temperature (RT) and then incubated overnight at 4 °C with the following primary antibodies: anti‐TPRA1 (Origene#TA334080), anti‐HA (Cell Signaling Technology#3724), anti‐β‐Tubulin (Abcam#ab6046), anti‐β‐Actin (Affinity#AF7018), and anti‐GAPDH (Proteintech#60004‐1‐Ig). The MXRA8 antibody was produced as previously described.^[^ ^23^ ^]^ After three washes with TBST (TBS containing 0.1% Tween‐20), the membranes were incubated with secondary antibodies (goat anti‐mouse IgG/HRP, Affinity #S0002; or goat anti‐rabbit IgG/HRP, Affinity #S0001) for 1 h at RT. Following another round of washes, the blots were developed using Immobilon Western HRP Substrate (Millipore#WBKLS0500) and visualized using a ChemiDoc XRS+ System (Bio‐Rad).

### Plasmid Construction

cDNA encoding TPRA1 (NM_0 011 36053) was purchased from GeneCopoeia. Full‐length TPRA1 tagged at the N‐ or C‐terminus with HA was cloned into the pLenti‐CMV‐BSD vector (a gift from G. Zhang) at the BamHI and PstI sites. Glycosylation site mutants (N11Q, N23Q) and a C‐terminal deletion mutant (Δ C‐tail, residues 286–373) were generated by PCR‐mediated mutagenesis. Constructs were confirmed by Sanger sequencing.

### Trans‐Complementation and Ectopic Expression

Lentiviral particles were produced by co‐transfecting HEK293T cells with the above‐constructed plasmid, pMD2.G, and pCMVR8.74. Cells, including HEK293, UMUC3, 22Rv1, and 22Rv1ΔTPRA1, were transduced with the lentivirus and selected with blasticidin for one week to generate stable overexpression cell lines. For infection studies, cells were inoculated with OVM‐GFP and analyzed by flow cytometry for GFP positivity.

### Flow Cytometry Analysis of HA‐Tagged TPRA1

Cells ectopically expressing HA‐tagged TPRA1 or Δ C‐tail were confirmed using flow cytometry. For surface staining of N‐terminally HA‐tagged proteins, cells were harvested using trypsin, washed with PBS, and incubated with an anti‐HA antibody (Cell Signaling Technology#3724) at 4 °C for 1 h. After washing, cells were stained with a goat anti‐rabbit IgG (H + L) secondary antibody conjugated to Alexa Fluor 488 (Thermo Scientific) for 30 min at room temperature. Following two additional washes, cells were analyzed by flow cytometry. For intracellular staining of C‐terminally HA‐tagged proteins expressed in 22Rv1ΔTPRA1 cells, a fixation and permeabilization step was included prior to incubation with the anti‐HA antibody.

### Plaque Formation Assay

UMUC3 cells were seeded in 96‐well plates and allowed to adhere overnight. OVM was added for 3 h, after which residual virus was removed by washing in PBS. A semisolid medium containing 0.75% agarose was then overlaid. At 48 h post‐infection, GFP‐positive plaques were imaged with Operetta CLS High Content Analysis System (PerkinElmer) and quantified using ImageJ.

### Genomic RNA Transfection and Analysis

To evaluate the effects of TPRA1 on OVM replication, capped viral genomic RNA was transfected into UMUC3 cells. Capped genomic RNA was generated from the XbaI‐linearized OVM cDNA clone using the RiboMAX Large Scale RNA Production System (Promega#P1280) according to the manufacturer's instructions. The purified RNA was transfected into cells using Lipofectamine MessengerMax reagent (Thermo Fisher Scientific#LMRNA003) following the manufacturer's protocol. Cells were incubated in medium containing 15 mm NH_4_Cl to prevent secondary rounds of infection. At 48 h post‐transfection, cells were harvested using trypsin, and GFP expression levels were analyzed by flow cytometry.

### Virus Binding and Internalization Assays

Cells were seeded in 6‐well plates and incubated overnight. For binding assays, OVM was incubated with the cells at 4 °C for 1 h. After three washes with ice‐cold PBS, cells were collected for RNA extraction and subsequent qPCR analysis. For internalization assays, following the 4 °C incubation, the cell‐virus mixture was transferred to 37 °C for 1 h. Cells were washed once with PBS and then treated with 500 ng mL^−1^ proteinase K on ice to remove non‐internalized virus particles. After three additional washes, cells were collected for RNA extraction. Viral abundance was quantified by qPCR.

### Immunofluorescence Staining and Confocal Microscopy

Cells were seeded overnight onto Lab‐Tek glass chambers (Thermo Scientific#155 411). After washing with PBS, cells were either treated or fixed with 4% paraformaldehyde for 15 min. Following three washes, cells were permeabilized with 0.1% Triton X‐100. Cells were then incubated overnight at 4 °C with primary antibodies diluted in Antibody Diluent (Agilent#S3022). The primary antibodies used were as follows: anti‐HA (Cell Signaling Technology#3724, 1:1600), anti‐EEA1 (Abcam#ab70521, 1:1000), anti‐Rab7 (Abcam#ab50533, 1:230), and anti‐LAMP1 (Proteintech#65051‐1, 1:500). After three washes with PBS, cells were incubated for 1 h with fluorescently tagged secondary antibodies: Alexa Fluor 488 anti‐mouse or Alexa Fluor 555 anti‐rabbit (Thermo Scientific, 1:500). Cells were then washed three times and incubated for 15 min with Hoechst 33 342 (1:1000), Alexa Fluor 488‐Wheat Germ Agglutinin (WGA‐AF488, Invitrogen#W11261, 1:1000), or iFluor 647‐Wheat Germ Agglutinin Conjugate (AAT Bioquest#25 559, 1:400). After three washes with PBS, cells were visualized using a Nikon Eclipse Ti confocal microscope. For visualizing OVM, the purified virus was labeled with EZ‐Link NHS‐PEG12‐Biotin (Thermo Scientific#A35389) according to the manufacturer's instructions. Unreacted biotin was removed using Zeba Spin Desalting Columns (Thermo Scientific#A57761). Cells were treated with biotin‐labeled OVM at 4 °C for 1 h, followed by incubation at 37 °C for 5 min. After three washes with PBS, immunofluorescence staining was performed using Streptavidin/Alexa Fluor 488 (Solarbio#K1068R‐AF488, 1:500) to visualize OVM.

### Pseudotyped Virus Experiments

The structural protein genes of OVM or Semliki Forest virus (SFV; NC_0 03215.1: 7420–11181) were cloned into the pcDNA3.1 vector. The vesicular stomatitis virus glycoprotein (VSV‐G) was encoded by the pMD2.G plasmid. The plasmid was co‐transfected with a GFP‐reporter lentiviral vector and pCMVR8.74 into HEK293T cells to generate pseudoviruses. At 24 h post‐transfection, pseudovirus‐containing supernatants were harvested and used to infect target cells. Pseudovirus entry efficiency was assessed at 48 hpi (VSV), 4 days (OVM) or 5 days (SFV) post‐infection by quantifying GFP expression via flow cytometry.

### Generation and Purification of Recombinant Proteins

Recombinant Proteins of a human Fc fragment with or without the extracellular domain of TPRA1 (residues 1–47) were expressed by GenScript using the CHO eukaryotic expression system. Briefly, DNA encoding the target protein was synthesized and sub‐cloned into pcDNA3.4. Transfection‐grade plasmids were prepared. CHO‐S cells were grown in serum‐free Expression Medium and maintained in Erlenmeyer Flasks at 37 °C with 5% CO_2_ on an orbital shaker. The recombinant plasmid encoding the target protein was transiently transfected into suspension CHO‐S cell cultures. The cell culture supernatant was collected for purification when the cell viability was less than 80%. Cell culture broth was centrifuged and followed by filtration. Filtered cell culture supernatant was loaded onto an affinity purification column. After washing and elution with appropriate buffers, the eluted fractions were pooled and buffer exchanged to the final formulation buffer (PBS). The purified protein was analyzed by SDS‐PAGE and SEC‐HPLC analysis to determine the molecular weight and purity.

### ELISA‐Based Binding Assays

Purified virions were immobilized at 1×10^6^ TCID50/well onto Nunc Immuno‐plates (Thermo Fisher #446 490) at 37 °C for 2 h. After washing three times in PBST (PBS with 0.1% Tween‐20) and blocking with 5% BSA, serial dilutions of TPRA1‐ECD‐Fc or Fc were added and incubated at 4 °C overnight. Plates were washed in PBST and incubated with HRP‐conjugated goat anti‐human IgG(H+L) (Proteintech #SA00001‐17, 1:1000) for 1 h at room temperature. After final washes, color was developed with 4,4′‐Diamino‐3,3′,5,5′‐tetramethylbiphenyl (Solarbio) and 1 m H_2_SO_4_. Absorbance was read at 450 nm using a microplate reader (BioTek). For deglycosylation, proteins were treated with Rapid PNGase F (NEB #P0711) according to the manufacturer's instructions. Deglycosylation was confirmed by SDS‐PAGE before use in ELISA.

### Biolayer Interferometry (BLI)

Binding kinetics were determined using the ForteBio Octet RED96e system (Sartorius). Aminopropylsilane (APS) biosensors (Sartorius #18‐5045) were loaded with OVM in PBS for 540 s, followed by a 420‐second wash in PBS. Association of TPRA1‐ECD‐Fc or control Fc (8 µm) was monitored for 1200 s in PBST buffer (PBS containing 0.02% Tween‐20 and 1 mg mL^−1^ BSA), and dissociation was monitored for 600 s in PBST‐BSA. For concentration‐dependent analysis, TPRA1‐ECD‐Fc was tested over a range of 8, 4, 2, 1, and 0.5 µm using the same experimental setup. Non‐specific interactions were monitored by applying the identical experimental setup to empty APS sensors (PBS during the loading step). Sensorgrams were fitted to a 1:1 binding model using ForteBio Data Analysis HT software to derive k_on_, k_off_, and K_d_ values.

### Glycan Array Analysis

Glycan binding specificity was profiled using the Glycan Array (RayBio #GA‐Glycan‐300‐1) according to the manufacturer's protocol. Briefly, OVM virus (Virotech) as well as E1 and E2 proteins recombinantly expressed in the bacterial system were purified, dialyzed, and then labeled with biotin. The Glycan Array glass slide was blocked with the provided blocking buffer. Protein samples of the same concentration were added to each well and incubated for 3 h. After washing to remove unbound protein, the array was incubated with Cy3‐conjugated Streptavidin for one hour. Following a final wash series, the slide was dried by centrifugation and immediately scanned using InnoScan 300 Microarray Scanner (Innopsys). Fluorescence intensity data for each glycan spot were extracted using the RayBio Analysis Tool.

### Tracking Cell Surface TPRA1

Cells overexpressing HA‐tagged TPRA1 (tagged at the extracellular N‐terminus) were incubated at 4 °C with an anti‐HA antibody (Cell Signaling Technology#3724; 1:400 dilution) for 1 h to label surface‐localized HA‐tagged proteins. Cells were washed five times with PBS to remove unbound antibody and then shifted to 37 °C for specific time points (2, 10, 30, and 60 min) to initiate endocytosis. At each time point, cells were fixed with 4% paraformaldehyde for 15 min. Subsequent immunofluorescence staining was performed to visualize TPRA1 intracellular trafficking.

### Tracking TPRA1 Recycling

We investigated TPRA1 recycling using an established antibody‐based recycling assay.^[^ ^73^ ^]^ Briefly, HA‐TPRA1‐expressing cells were incubated with anti‐HA antibody (1:400) and allowed to internalize into the cells by incubation at 37 °C for 30 min. After washing with PBS to remove unbound antibody, the remaining surface‐bound HA antibody was blocked with an unconjugated secondary antibody (Proteintech#B900210) at 4 °C for 1 h. Cells were subsequently incubated at 37 °C for the indicated times (0, 15, and 30 min) to allow internalized HA‐TPRA1 to return to the cell surface. For immunofluorescence detection, cells were fixed at each time point with 4% paraformaldehyde for 15 min. Subsequent immunofluorescence staining was performed to visualize recycled TPRA1. For flow cytometry, cells were incubated with Alexa Fluor 488 anti‐rabbit antibody (Thermo Scientific, 1:500) and analyzed by flow cytometry.

### Co‐Immunoprecipitation (Co‐IP) and Mass Spectrometry (MS)

UMUC3 cells overexpressing HA‐tagged TPRA1 or ΔC‐tail were collected and lysed on ice for 30 min using cell lysis buffer (Beyotime#P0013). Anti‐HA magnetic beads (MedChemExpress#HY‐K0201) were prepared according to the manufacturer's protocol. After removing cell debris by centrifugation, clarified lysate supernatants were incubated with the beads overnight at 4 °C. Following a series of washes with TBST, a small aliquot of beads was resolved by SDS‐PAGE to confirm the presence of HA‐tagged proteins. The remaining beads were subjected to Liquid Chromatography‐Tandem Mass Spectrometry (LC‐MS/MS; Wininnovate Bio) to identify co‐immunoprecipitated proteins.

### Arbidol Inhibition Assay

Arbidol (MedChemExpress #HY‐14904) was dissolved in DMSO to prepare a 10 mm stock solution. TPRA1 or vector‐expressing cells were seeded in 24‐well plates and allowed to adhere overnight. Cells were pre‐treated with either DMSO (vehicle control) or 10 µm Arbidol (diluted from the stock in complete medium) for 3 h at 37 °C. Subsequently, 0.1 MOI of OVM‐GFP was added into wells. After 28 h of incubation, infection efficiency was quantified by measuring the percentage of GFP‐positive cells via flow cytometry and was corroborated by fluorescence microscopy imaging.

### siRNA Screens

SiRNA library targeting 99 candidate genes was purchased from Ribobio (Guangzhou, China). For each gene, a pool of three distinct siRNA duplexes was transfected into cells at a final concentration of 30 nm. About 44 h post‐transfection, cells were challenged with OVM at the indicated MOI. Cell viability was assessed 48 hpi using Cell Counting Kit‐8 (TargetMol #C0005).

### Viral Titer Assay

BHK‐21 cells were seeded in 96‐well plates at 1500 cells per well and incubated overnight. Virus‐containing supernatants were serially diluted in MEM medium supplemented with 10% fetal bovine serum (FBS) and added to the BHK‐21 cells. At 72 h post‐infection, GFP expression and cytopathic effect (CPE) were assessed using a fluorescence microscope. Viral titers were calculated using the Spearman‐Karber method.

### Cell Viability Assay

Cell viability was assessed using the Cell Counting Kit‐8 (TargetMol #C0005) following the manufacturer's instructions. In brief, cells were seeded into multi‐well plates and treated as indicated. After treatment, 10 µL of the CCK‐8 solution was added to each well. The plates were then incubated at 37 °C for 1–4 h. Absorbance was measured at 450 nm using a microplate reader (BioTek).

### Quantitative RT–PCR of OVM RNA

RNA was extracted from samples using TRIzol (Invitrogen) and quantified with a NanoDrop spectrophotometer (Thermo Fisher). OVM genomic RNA was measured using a specific probe (CTCTCATCAGCAGCGAGCCTCCT) and primers (Q3S1‐F: GGGATTCACTACACCTGCTTAGAC, Q3S1‐R: GCTGACTCTGTCTGCGTAACC) with the FastKing One Step RT‐qPCR Kit (TIANGEN #FP314). Results were reported as genome equivalents per microgram of total RNA.

### Mouse Experiments

All animal experiments were conducted in accordance with protocols approved by the Institutional Animal Care and Use Committee (IACUC) of Sun Yat‐sen University (2 024 001 778). Mice were purchased from Guangdong GemPharmatech Co., Ltd. and maintained under specific‐pathogen‐free (SPF) conditions. For the xenograft model, 2 × 10⁶ vector or TPRA1‐overexpressing UMUC3 cells, or 5 × 10^6^control or ΔTPRA1 PC‐9 cells were subcutaneously inoculated into the hind flanks of 6‐week‐old female BALB/c‐nu/nu mice. After palpable tumors developed (50–150 mm^3^), mice were randomized to receive corresponding treatments. UMUC3 tumor‐bearing mice received daily tail vein injections of 300 µL PBS or OVM (1×10^7^ TCID50/mL) for 7 days. PC9 tumor‐bearing mice received intratumoral injections of 50 µL PBS or OVM (1×10^7^ TCID50/mL) once weekly. Tumor volume was measured every 2–3 days using the formula: Tumor Volume = (length×width^2^)/2. Mice were euthanized when tumors exceeded 2000 mm^3^. At 10 days (UMUC‐3 model) or 23 days (PC‐9 model) after the first virus injection, tumors were harvested, homogenized in TRIzol reagent using a mechanical homogenizer, and total RNA was extracted for virus genome detection.

For Syngeneic Tumor Model, A20, CT26, EMT6, H22, or Renca cells were implanted into BALB/c mice, while B16BL6, B16F10, Hepa1‐6, LLC, MC38, Pan02, or RM1 cells were implanted into C57BL/6 mice. Mice received daily tail vein injections of 200 µL PBS or OVM (1×10^7^ TCID50/mL) for 5 days. Tumor growth inhibition (TGI) was calculated as TGI = (1−TmRTV/ CmRTV)×100%), where TmRTV (treatment group) and CmRTV (control group) represent mean relative tumor volumes (RTV), calculated as RTV = V_t_/V_0_, with V_0_ = baseline mean volume and V_t_ = volume at each time point. The maximum inhibition rate was used for correlation analysis between TPRA1 expression and tumor growth inhibition.

### Ex Vivo Anticancer Activity

The ex vivo anticancer activity of OVM was evaluated as previously described.^[^ ^14^ ^]^ Patient‐derived tissue specimens were obtained from patients with informed consent during tumor resection. This study was approved by the ethics review committee of Sun Yat‐sen University (2 024 042). Tumor samples were aseptically dissected into ≈1 mm^3^ fragments using a scalpel and placed in 24‐well plates containing 1.5 mL DMEM supplemented with 15% FBS. After 24 h of culture at 37 °C with 5% CO_2_, baseline viability (A score) was quantified using the tissue culture‐endpoint staining‐computer image analysis (TECIA) method based on the volumetric integral of the explants. Samples were treated with OVM (4.61 × 10^7^ TCID50) or PBS control for 3 days. After treatment, 100 µL of MTT solution (3‐(4,5‐dimethylthiazol‐2‐yl)‐2,5‐diphenyltetrazolium bromide, 5 mg mL^−1^) was added to each well and incubated for 4 h at 37 °C. Post‐staining viability (B score) was determined by analyzing the blue‐stained area and staining intensity using the same system. Percentage inhibition was calculated as inhibition (%) = [1−(mean of B scores of treated sample/mean of A scores of treated sample)/(mean of B scores of control)/(mean of A scores of control)]×100%. Each treatment was tested in triplicate for every sample.

### Pan‐Cancer Analysis of TPRA1 Expression

RNA sequencing data for TPRA1 expression in human tumor tissues and matched non‐tumor tissues were obtained from The Cancer Genome Atlas (TCGA) and analyzed as FPKM (Fragments Per Kilobase of transcript per Million mapped reads) values within the Sangerbox platform (http://sangerbox.com/). For tumor types with insufficient matched healthy tissues (≤5 samples) in TCGA, non‐tumor tissues from TCGA and the Genotype‐Tissue Expression (GTEx) project were pooled and analyzed using TPM (Transcripts Per Million) values.

### Tissue Microarray

Tissue microarrays (TMA) were purchased from Shanghai Outdo Biotech Co., Ltd. All TMAs derived from human tissue samples were collected with informed consent and approved by the Institutional Ethics Review Committee of Shanghai Outdo Biotech under ethical approval numbers SHYJS‐CP‐1604004, SHYJS‐CP‐1610001, and SHYJS‐CP‐1907005. Each TMA core measured 1.5 mm in diameter, and sections were cut to a thickness of 4 µm. Immunohistochemistry (IHC) staining was performed on the TMAs using a TPRA1‐specific antibody custom‐developed by Abcam and generously provided by Guangzhou Virotech Pharmaceutical Technology Co., Ltd. Following staining, the TMA slides were scanned using the NanoZoomer S360 digital slide scanner (Hamamatsu Photonics). The staining intensity of TPRA1 was analyzed using NDP.view 2 software.

### Statistical Analysis

Data are expressed as mean ± standard deviation (SD). Statistical analyses were performed using GraphPad Prism 9. For comparisons between two groups, an unpaired Student's *t*‐test was used. For comparisons involving three or more groups, one‐way analysis of variance (ANOVA) was applied. Tumor volumes were analyzed using one‐way ANOVA at the terminal endpoint. Survival differences were compared using the log‐rank test. Peason correlation coefficient was used to determine statistical dependence. A *P*‐value < 0.05 was considered statistically significant, with the following thresholds ^*^ *P* < 0.05, ^**^ *P* < 0.01, ^***^ *P* < 0.001, and ^****^ *P* < 0.0001.

## Conflict of Interest

The authors declare no conflict of interest.

## Supporting information



Supporting Information

## Data Availability

The data that support the findings of this study are available from the corresponding author upon reasonable request.;
