# Supplementary material for: Identification of TPRA1 as a Novel Receptor and Predictive Biomarker for Oncolytic Virus M1
Source: Adv Sci (Weinh). 2025 Oct 6;12(48):e10597. doi: 10.1002/advs.202510597 (PMC12752648; doi:10.1002/advs.202510597)
Supplement: Supplementary file 1 — Supporting Information [file ADVS-12-e10597-s001.docx]

Supporting Information

Identification of TPRA1 as a Novel Receptor and Predictive Biomarker for Oncolytic Virus M1

Linyi Hu, Guigen Zhang, Yuan Lin, Shiming Yi, Jingyu Yang, Xueying Lin, Deli Song, Zhiheng Liu, Jiayu Zhang, Ying Zeng, Shanyu Huang, Zhen Fan, Jifu Zhang, Ying Liu, Cheng Hu, Wenbo Zhu, Wei Yin, Jun Hu, Guangmei Yan, Jing Cai* and Jiankai Liang*


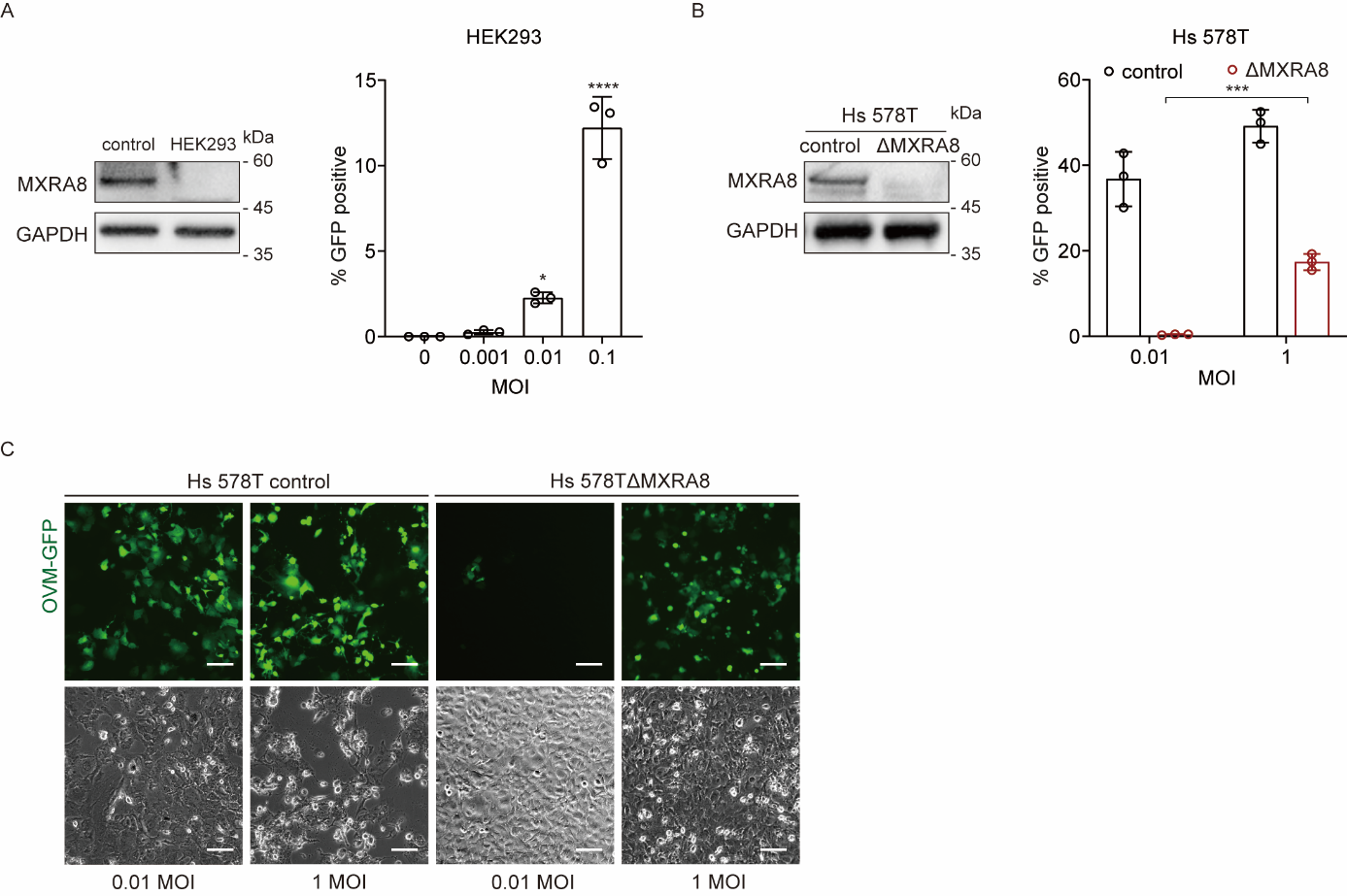


**Figure S1.** Cells Lacking MXRA8 Are Susceptible to OVM Infection. **A)** Immunoblotting of MXRA8 and GAPDH in control and HEK293 cells (left). GFP-positive cell rates following OVM-GFP infection at different MOIs were quantified at 48 h post-infection (right). Data are presented as the percentage of GFP-positive cells. **B)** Immunoblotting of MXRA8 and GAPDH in control and ΔMXRA8 Hs 578T cells (left). GFP-positive cell rates following OVM-GFP infection at different MOIs were quantified at 28 h post-infection (right). Data are presented as the percentage of GFP-positive cells. **C)** Phase-contrast and fluorescence microscopy images of Hs 578T control and ΔMXRA8 cells infected with OVM-GFP at MOIs of 0.01 and 1. Scale bars: 100 μm. The data are representative of three experiments. *P* values were determined by Student’s *t*-test or one-way ANOVA. **P* < 0.05; ****P* < 0.001; and *****P* < 0.0001.


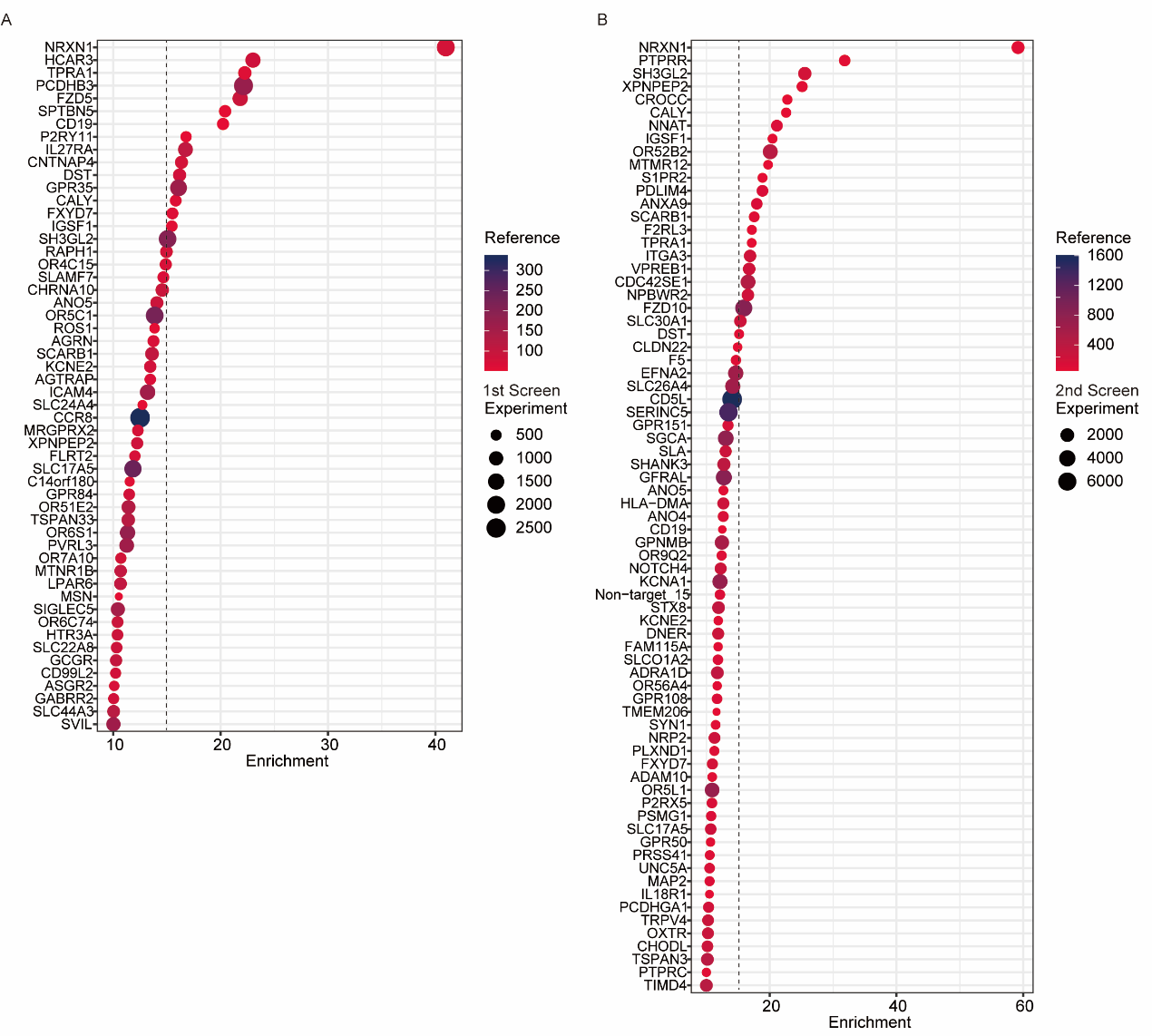


**Figure S2.** CRISPR-Cas9 Screen Results. **A)** Genes enriched in the first (1st) CRISPR screen. **B)** Genes enriched in the second (2nd) CRISPR screen. Genes with sgRNAs read counts > 50 in the reference library and enrichment > 15-fold after the screen were considered as enriched genes. The dotted line indicates the 15-fold enrichment threshold.


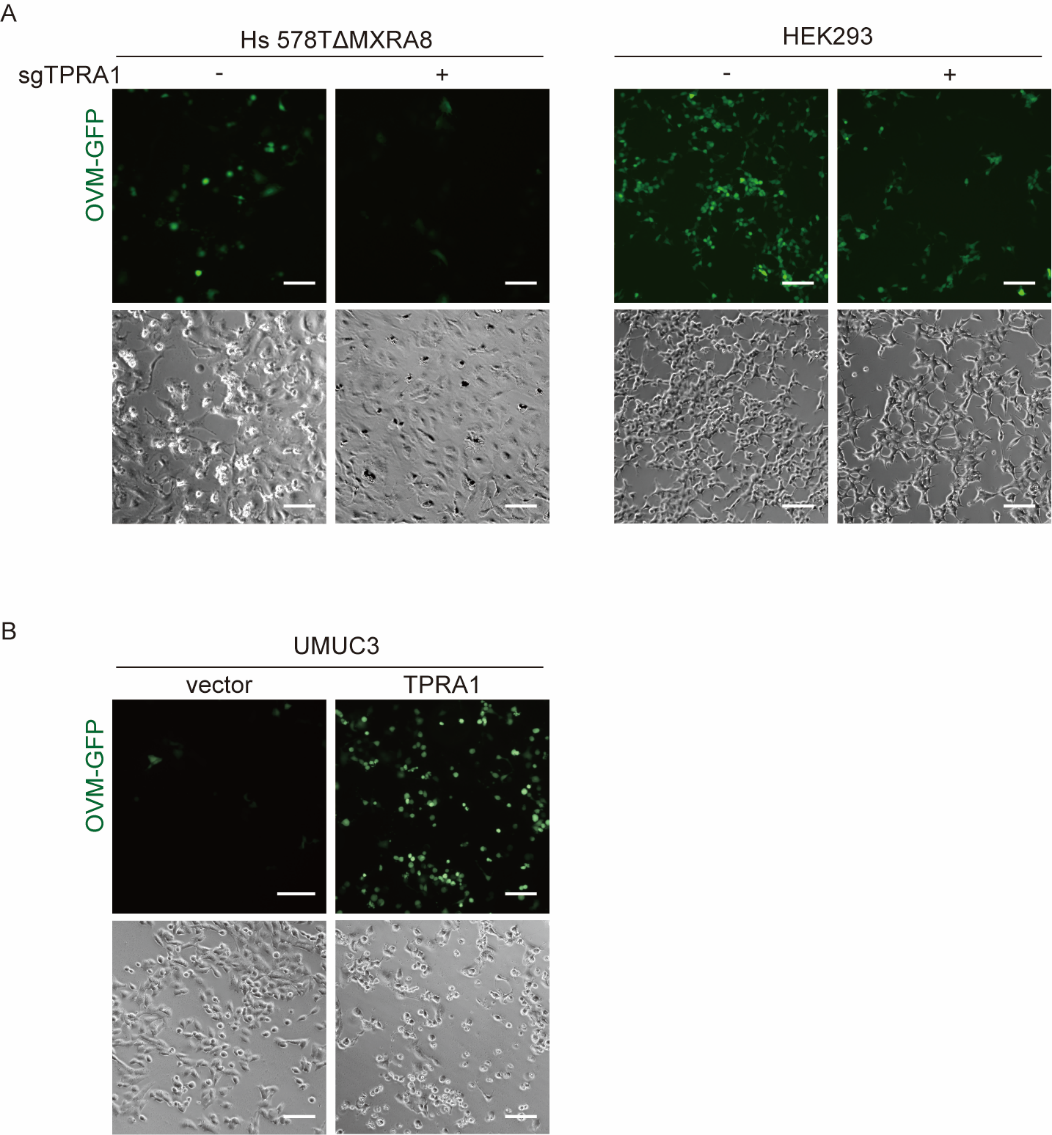


**Figure S3.** TPRA1 is a Critical Factor for OVM Infection. **A)** Microscopy images of HEK293 and Hs 578T cells as described in Figure 1C.**B)** Microscopy images of UMUC3 cells as described in Figure. 1F. Scale bar: 100 μm. The data are representative of three experiments.


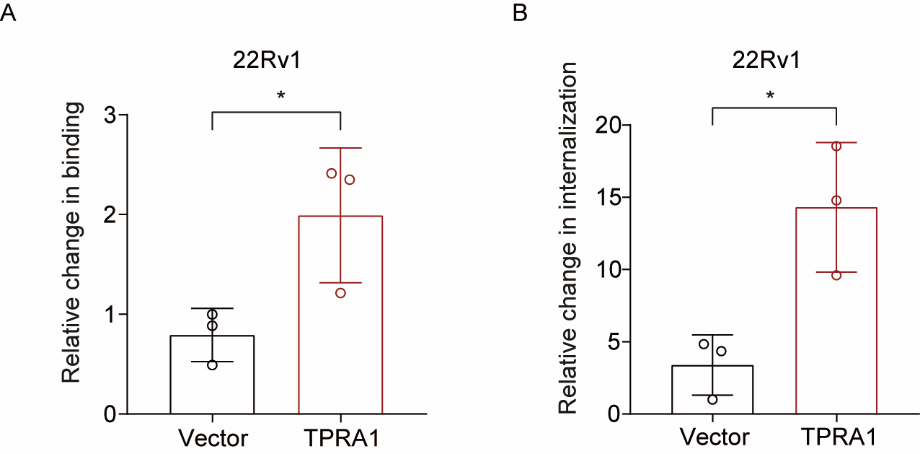


**Figure S4.** TPRA1 enhances OVM binding and attachment. **A)** 22Rv1 cells overexpressing TPRA1 or vector were incubated with OVM at 4 °C for 1 h. Cells were then collected, and OVM RNA was quantified by qRT-PCR using β-Actin as a control. **B)** 22Rv1 cells overexpressing TPRA1 or vector were incubated with OVM at 4 °C for 1 h and shifted to 37 °C for another 1 h. Cells were collected and analyzed by qRT-PCR for OVM RNA and β-Actin. The data are representative of three experiments. *P* values were determined by Student’s *t*-test. **P* < 0.05.

**
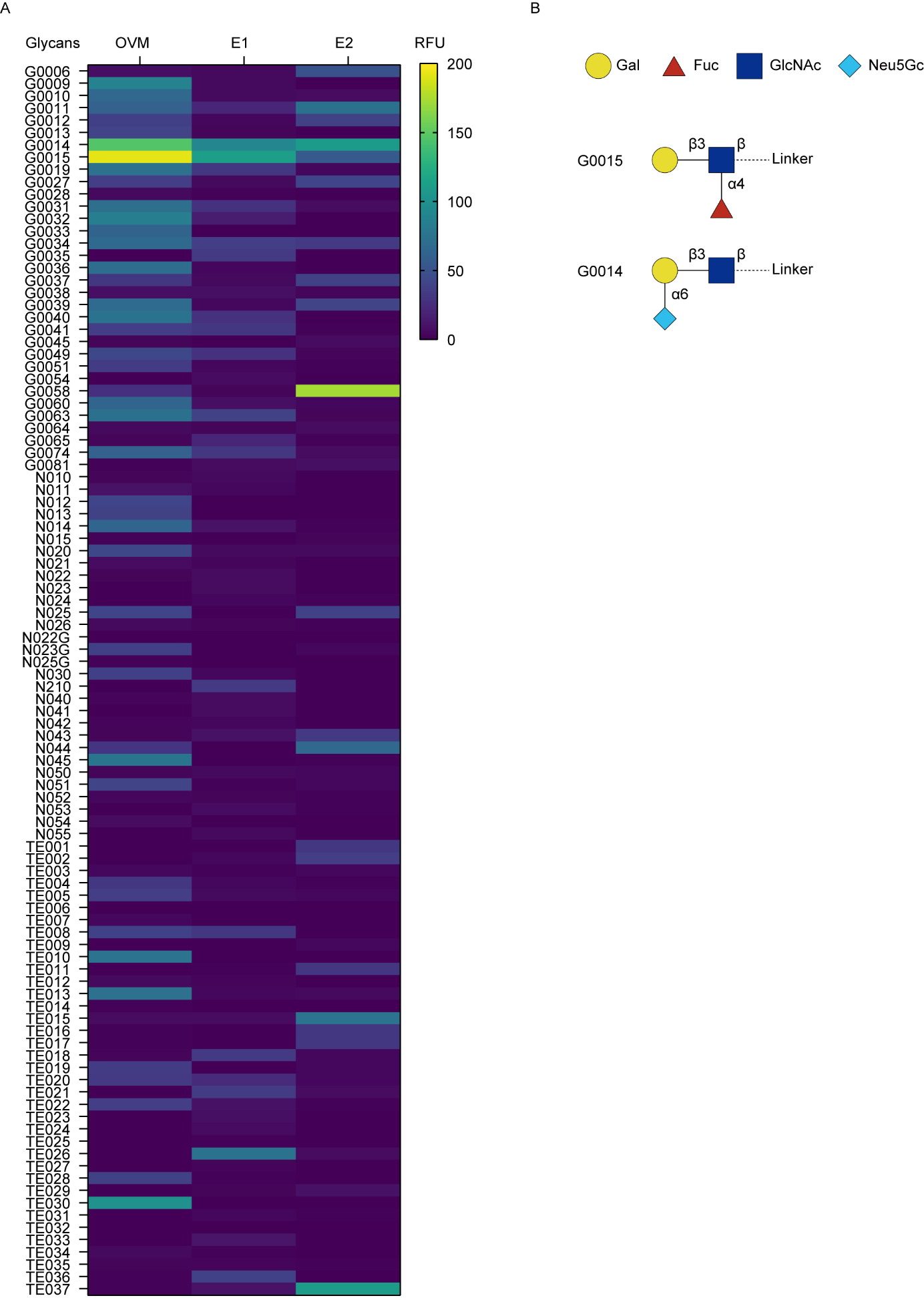
**

**Figure S5.** OVM and its envelope proteins bind glycans. **A)** Glycan array analysis of OVM, E1 and E2 proteins binding to 100 immobilized glycans. RFU, relative fluorescence units. **B)** Structure of G0015 glycan (Gal-β-1,3-(Fuc-α-1,4)-GlcNAc-β-[LewisA]–Sp) and G0014 glycan (Neu5Gc-α-2,6-Gal-β-1,3-GlcNAc-β-Sp).


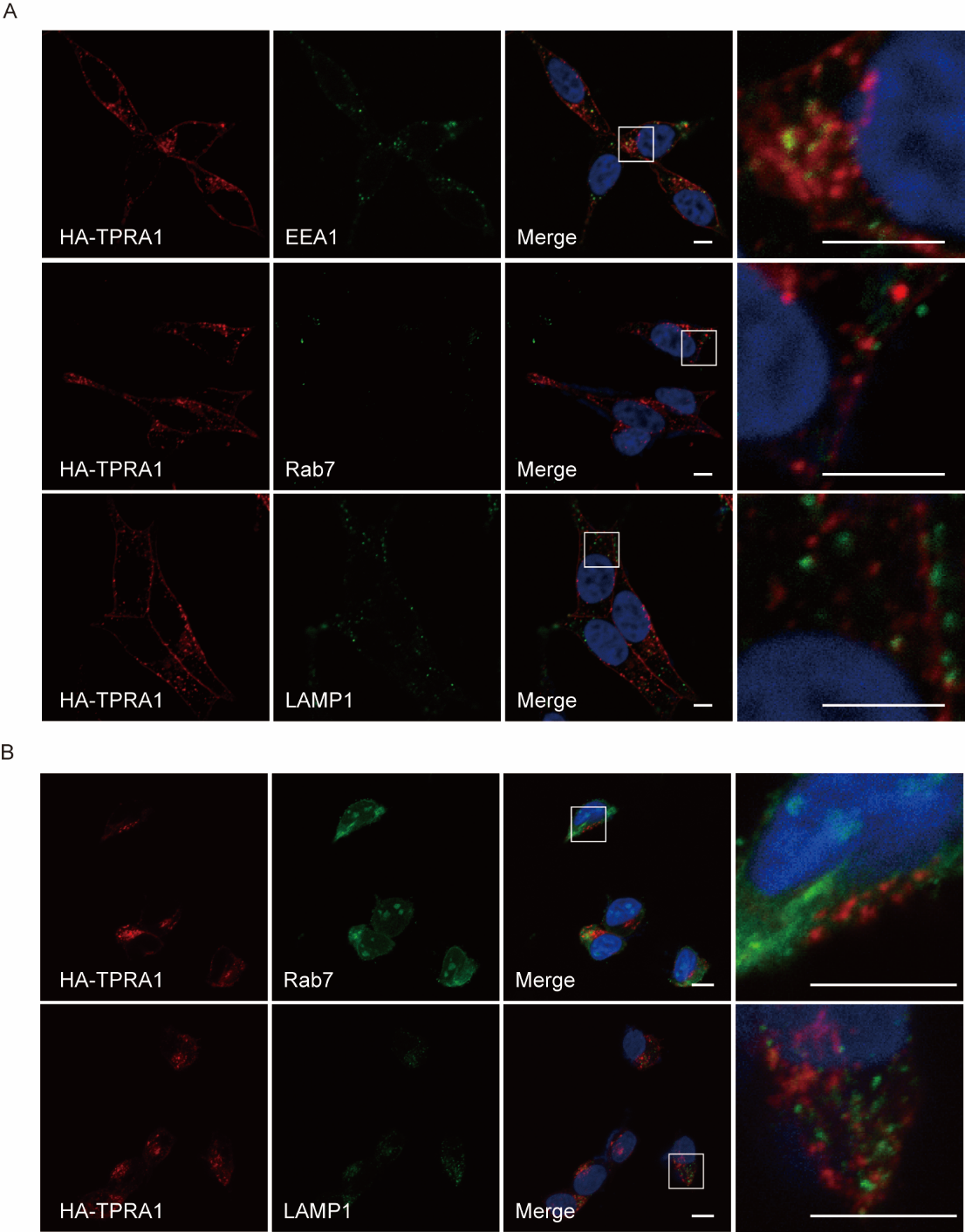


**Figure S6.** TPRA1 Subcellular Localization. **A)** Confocal immunofluorescence microscopy of ectopically expressed HA-TPRA1 in 22Rv1 cells shown with early endosome marker EEA1, late endosome marker Rab7, or lysosome marker LAMP1. Scale bar: 5 μm. **B)** Subcellular localization of ectopically expressed TPRA1-HA in UMUC3 cells shown with late endosome marker Rab7 or lysosome marker LAMP1. Scale bar: 10 μm. The data are representative of three experiments.


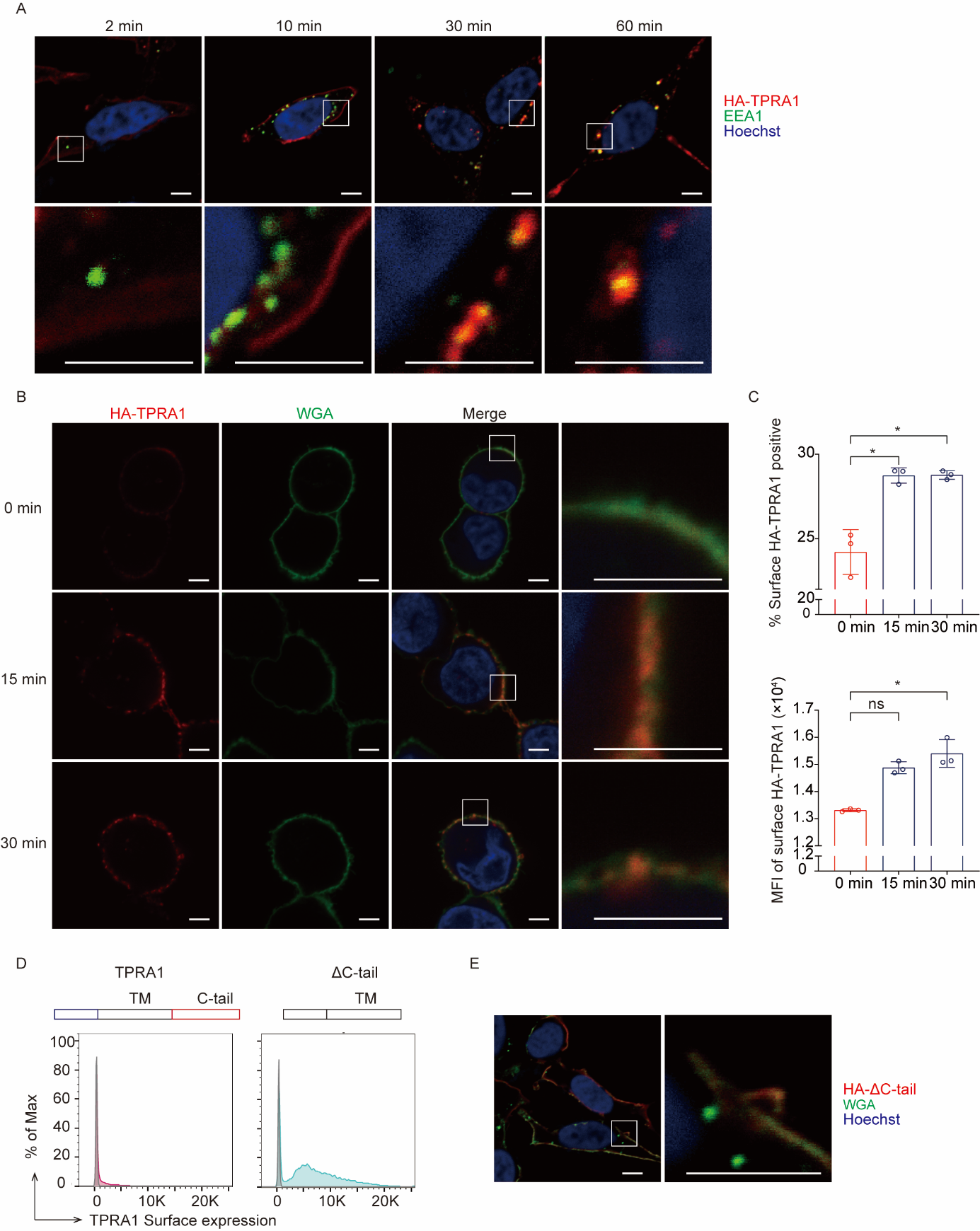


**Figure S7.** TPRA1 is a recycling receptor. **A)** Tracking of HA-TPRA1 endocytosis in 22Rv1 cells using anti-HA antibodies. Cells were incubated with anti-HA antibodies at 4 °C for 1 h, followed by incubation at 37 °C to initiate endocytosis. Cells were fixed at indicated time points, and internalized TPRA1 was visualized by confocal immunofluorescence. Scale bar: 5 μm. **B-C)** HA-TPRA1-expressing UMUC3 cells were incubated with anti-HA antibody at 37 °C. After removal of unbound antibody, residual surface-bound antibody was blocked with unconjugated secondary antibody at 4 °C. Cells were then incubated at 37 °C for the indicated times (0, 15 and 30 min) to allow internalized HA-TPRA1 return to the plasma membrane. Recycled HA-TPRA1 on the cell surface was subsequently detected using a fluorescently labeled secondary antibody. B) Confocal immunofluorescence microscopy of recycled HA-TPRA1 on the cell surface. Scale bar: 5 μm. C) Quantification of cell surface HA-TPRA1 by flow cytometry. Upper: percentage of surface HA-TPRA1-positive cells; Lower: mean fluorescence intensity of surface HA-TPRA1. **D)** 22Rv1 cells overexpressing HA-TPRA1 or HA-ΔC-tail were collected and inoculated with anti-HA antibodies to detect surface expression of HA-tagged proteins by flow cytometry. **E)** Confocal immunofluorescence microscopy of 22Rv1 cells expressing HA-tagged ΔC-tail. Green: Wheat germ agglutinin (WGA); blue: Hoechst; scale bar: 5 μm. The data are representative of three experiments. *P* values were determined by one-way ANOVA. ns, not significant; **P* < 0.05.


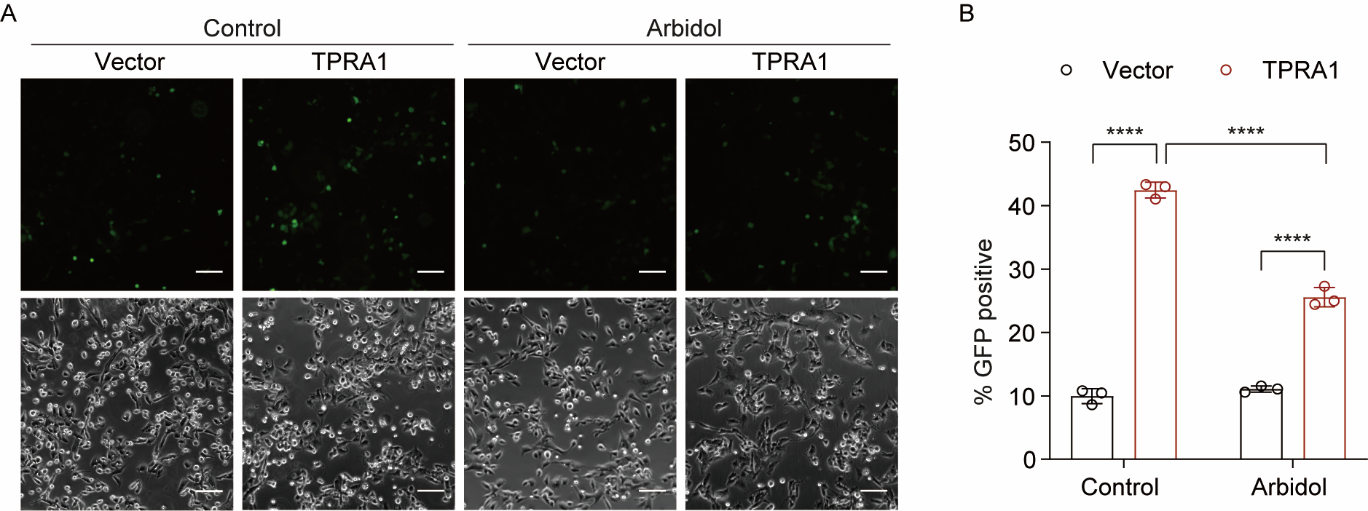


**Figure S8.** Arbidol impairs TPRA1-enhanced OVM infection. **A-B)** TPRA1 or vector-expressing UMUC3 cells were pretreated with 10 μM Arbidol or DMSO vehicle control for 3 h. Cells were then infected with OVM-GFP (0.1 MOI) for 28 h. A) Representative fluorescent microscopy images showing GFP-positive infected cells. Scale bar: 100 μm. B) Quantification of OVM infection by flow cytometry analysis of GFP-positive cells. The data are representative of three experiments. *P* values were determined by one-way ANOVA. *****P* < 0.0001.


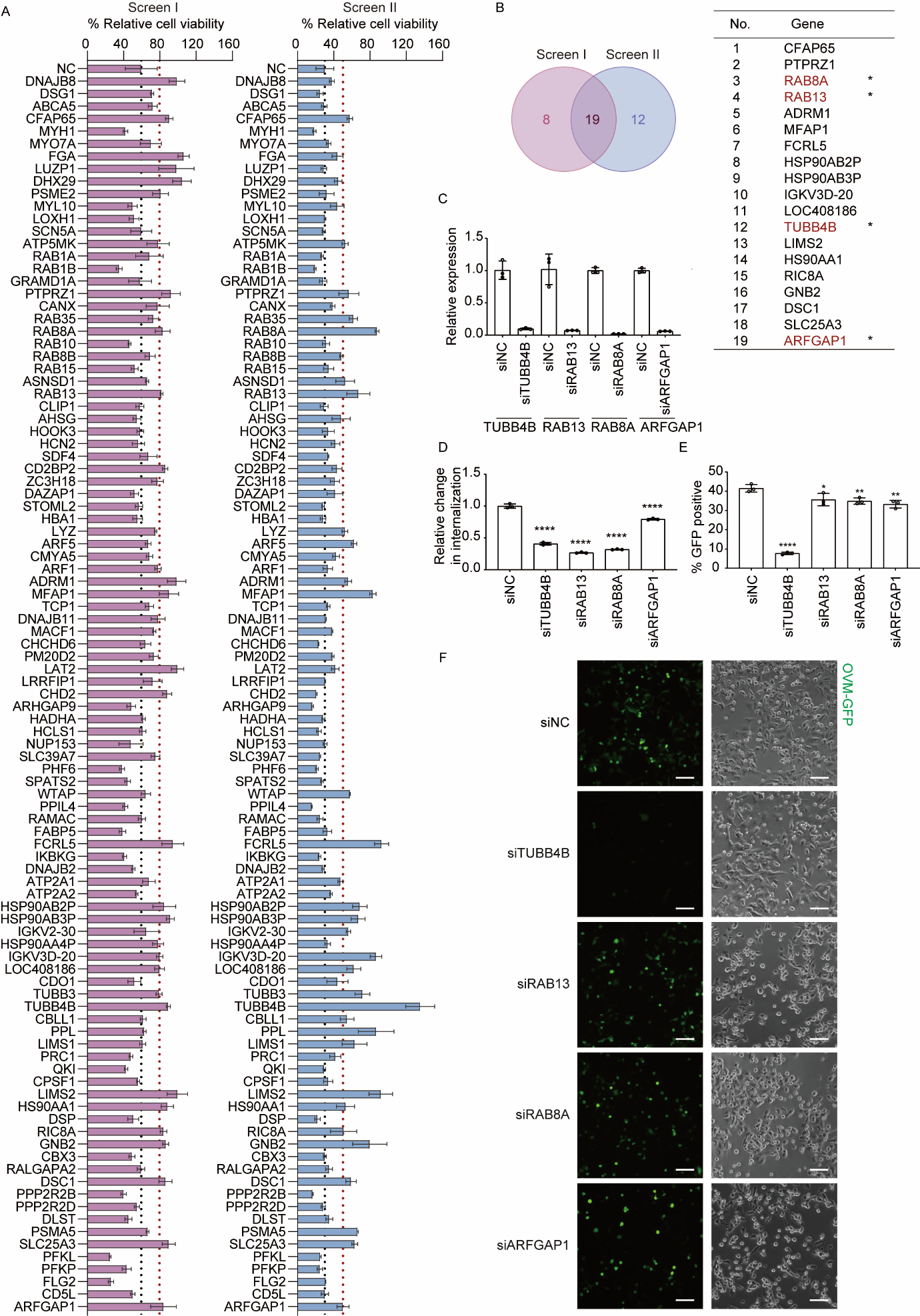


**Figure S9.** Identification and validation of TPRA1 C-tail interactors involved in OVM internalization. **A)** TPRA1-expressing UMUC3 cells were transfected with siRNAs targeting 99 candidate interacting genes or a non-targeting negative control siRNA (siNC), followed by OVM infection (2 MOI for Screen I and 4 MOI for Screen II). Cell viability was measured at 48 hpi. Black dotted line: viability of siNC. Red dotted line: viability 20% over siNC. **B)** 19 genes when knockdown reduced OVM killing efficacy for over 20% in both rounds of siRNA screens. Red: endocytosis-related genes. **C)** qPCR validation of knockdown efficiency for the endocytosis-related genes. **D)** OVM internalization after knocking down the endocytosis-related genes in UMUC3 cells. The data are presented as mean±SD from three technical replicates. **E)** OVM infection rate after gene knockdown. The data are representative of three experiments. *P* values were determined by one-way ANOVA. **P* < 0.05; ***P* < 0.01; and *****P* < 0.0001. **F)** Representative microscopic images of OVM-infected cells. Scale bars: 100 μm.


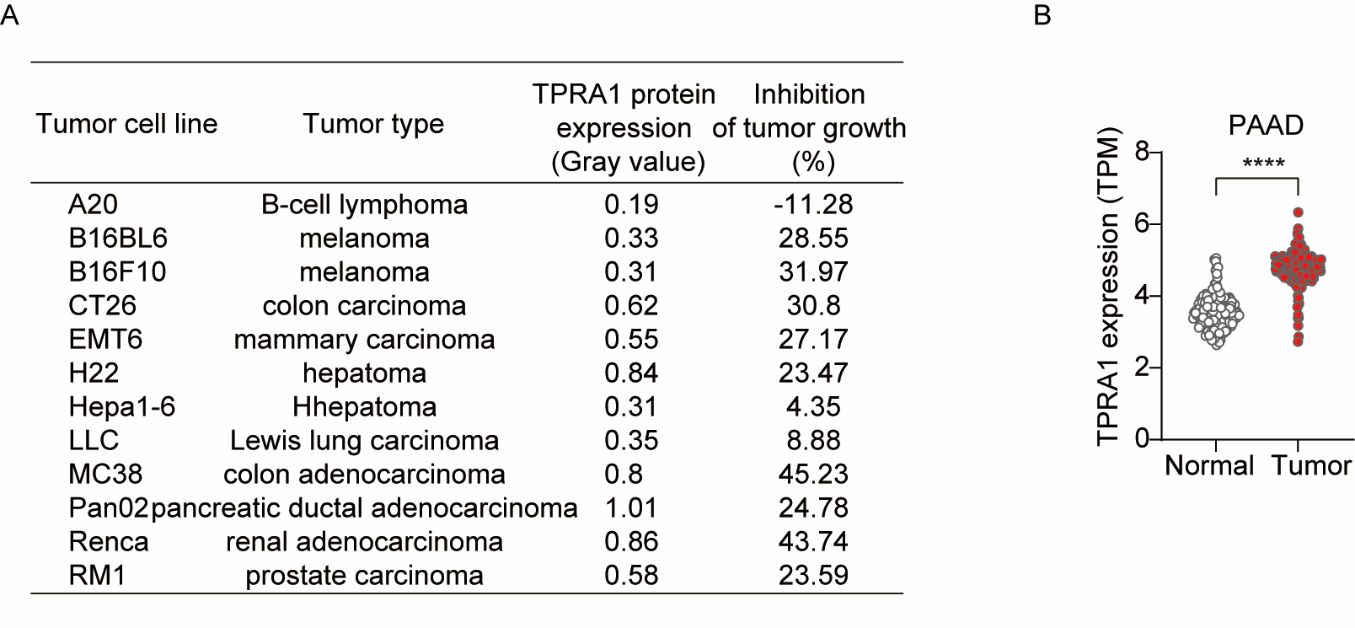


**Figure S10.** TPRA1 Expression and OVM Efficacy in Mouse Models, and TPRA1 Expression in Pancreatic Tumors. **A)** Maximum OVM inhibition rate in mouse-derived tumors and corresponding TPRA1 protein expression levels detected by Western blot. n=9 mice. **B)** TPRA1 mRNA expression levels in pancreatic adenocarcinoma (PAAD) compared to normal pancreatic tissue. The tumor tissue data were obtained from TCGA, while the normal tissue data were sourced from the GTEx project. TPM: Transcripts Per Million. *****P* < 0.0001.

**Supplementary Table 1.** Glycans tested for OVM binding.

| ID | Glycan |
| --- | --- |
| G0006 | β-GlcNAc-Sp |
| G0009 | Gal-β-1,3-GlcNAc-β-Sp |
| G0010 | Gal-α-1,3-Gal-β-1,3-GlcNAc-β-Sp |
| G0011 | Neu5Ac-α-2,3-Gal-β-1,3-GlcNAc-β-Sp |
| G0012 | Neu5Ac-α-2,6-Gal-β-1,3-GlcNAc-β-Sp |
| G0013 | Neu5Gc-α-2,3-Gal-β-1,3-GlcNAc-β-Sp |
| G0014 | Neu5Gc-α-2,6-Gal-β-1,3-GlcNAc-β-Sp |
| G0015 | Gal-β-1,3-(Fuc-α-1,4)-GlcNAc-β-[LewisA]–Sp |
| G0019 | GlcNAc-β-1,3-Gal-β-1,4-Glc-β-Sp |
| G0027 | GlcNAc-β-1,6-GlcNAc-β-Sp |
| G0028 | 4-P-GlcNAc-b-1,4-Man-b-Sp |
| G0031 | Gal-β-1,4-GlcNAc-β-Sp |
| G0032 | Gal-β-1,4-(Fuc-α-1,3)-GlcNAc-β-[LewisX]–Sp |
| G0033 | Neu5Ac-α-2,3-Gal-β-1,4-(Fuc-α-1,3)-GlcNAc-β-[SialylLewisX]-Sp |
| G0034 | Neu5Ac-α-2,3-Gal-β-1,3-(Fuc-α-1,4)-GlcNAc-β-[SialylLewisA]-Sp |
| G0035 | Neu5Gc-α-2,3-Gal-β-1,3-(Fuc-α-1,4)-GlcNAc-β-[SialylLewisA]-Sp |
| G0036 | Gal-α-1,4-Gal-β-1,3-GlcNAc-β-Sp |
| G0037 | Gal-β-1,4-GlcNAc-β-1,3-Gal-β-1,4-Glc-β-[LNnT]-Sp |
| G0038 | GlcA-β-1,4-GlcNAc-α-1,4-GlcA-β-Sp |
| G0039 | GlcNAc-β-1,6-(Gal-β-1,3)-GalNAc-α-O-Ser-Sp4 |
| G0040 | Neu5Ac-α-2,3Gal-β-1,4-(6S)GlcNAc-β-Sp |
| G0041 | GalNAc-β-1,4-GlcNAc-β-Sp2 |
| G0045 | GlcNAc-β-1,2-Man-α-Sp |
| G0049 | Gal-β-1,4-(Fuc-α-1,3)-GlcNAc-β-1,3-Gal-β-Sp1 |
| G0051 | GlcNAc-β-1,4-GlcNAc-β-Sp1 |
| G0054 | GlcNAc-α-1,3-(Glc-α-1,2-Glc-α-1,2)-Gal-α-1,3-Glc-α-Sp |
| G0058 | Fuc-α-1,2-Gal-β-1,4-GlcNAc-β-[BloodHantigentrisaccharide]-Sp1 |
| G0060 | Fuc-α-1,2-Gal-β-1,3-GlcNAc-β-1,3-Gal-β-1,4-Glc-β-[LNFPI]-Sp1 |
| G0063 | (Fuc-α-1,2)-Gal-β-1,4-(Fuc-α-1,3)-GlcNAc-β-[LewisY]-Sp1 |
| G0064 | (Fuc-α-1,2)-Gal-β-1,3-(Fuc-α-1,4)-GlcNAc-β-[LewisB]-Sp1 |
| G0065 | Gal-β-1,3-(Fuc-α-1,4)-GlcNAc-β-1,3-Gal-β-1,4-(Fuc-α-1,4)-Glc-β-[LewisA]-Sp1 |
| G0074 | Gal-α-1,4-Gal-β-1,4-GlcNAc-β-Sp1 |
| G0081 | GlcNAc-α-1,4-GlcA-β-1,4-GlcNAc-α1,4-GlcA-β-Sp |
| N010 | Man-α-1,6-(Man-α-1,3-)Man-α-1,6-(GlcNAc-β-1,2-Man-α-1,3-)Man-β-1,4-GlcNAc-β-1,4-GlcNAc-Sp5 |
| N011 | Man-α-1,6-(Man-α-1,3-)Man-α-1,6-(Gal-β-1,4-GlcNAc-β-1,2-Man-α-1,3-)Man-β-1,4-GlcNAc-β-1,4-GlcNAc-Sp5 |
| N012 | Man-α-1,6-(Man-α-1,3-)Man-α-1,6-(Neu5Ac-α-2,3-Gal-β-1,4-GlcNAc-β-1,2-Man-α-1,3-)Man-β-1,4-GlcNAc-β-1,4-GlcNAc-Sp5 |
| N013 | Man-α-1,6-(Man-α-1,3-)Man-α-1,6-(Neu5Ac-α-2,6-Gal-β-1,4-GlcNAc-β-1,2-Man-α-1,3-)Man-β-1,4-GlcNAc-β-1,4-GlcNAc-Sp5 |
| N014 | Man-α-1,6-(Man-α-1,3-)Man-α-1,6-[Gal-β-1,4-(Fuc-α-1,3-)GlcNAc-β-1,2-Man-α-1,3-]Man-β-1,4-GlcNAc-β-1,4-GlcNAc-Sp5 |
| N015 | Man-α-1,6-(Man-α-1,3-)Man-α-1,6-[Neu5Ac-α-2,3-Gal-β-1,4-(Fuc-α-1,3-)GlcNAc-β-1,2-Man-α-1,3-]Man-β-1,4-GlcNAc-β-1,4-GlcNAc-Sp5 |
| N020 | GlcNAc-β-1,2-Man-α-1,3-Man-β-1,4-GlcNAc-β-1,4-GlcNAc-Sp5 |
| N021 | Gal-β-1,4-GlcNAc-β-1,2-Man-α-1,3-Man-β-1,4-GlcNAc-β-1,4-GlcNAc-Sp5 |
| N022 | Neu5Ac-α-2,3-Gal-β-1,4-GlcNAc-β-1,2-Man-α-1,3-Man-β-1,4-GlcNAc-β-1,4-GlcNAc-Sp5 |
| N023 | Neu5Ac-α-2,6-Gal-β-1,4-GlcNAc-β-1,2-Man-α-1,3-Man-β-1,4-GlcNAc-β-1,4-GlcNAc-Sp5 |
| N024 | Gal-β-1,4-(Fuc-α-1,3-)GlcNAc-β-1,2-Man-α-1,3-Man-β-1,4-GlcNAc-β-1,4-GlcNAc-Sp5 |
| N025 | Neu5Ac-α-2,3-Gal-β-1,4-(Fuc-α-1,3-)GlcNAc-β-1,2-Man-α-1,3-Man-β-1,4-GlcNAc-β-1,4-GlcNAc-Sp5 |
| N026 | Gal-α-1,3-Gal-β-1,4-GlcNAc-β-1,2-Man-α-1,3-Man-β-1,4-GlcNAc-β-1,4-GlcNAc-Sp5 |
| N022G | Neu5Gc-α-2,3-Gal-β-1,4-GlcNAc-β-1,2-Man-α-1,3-Man-β-1,4-GlcNAc-β-1,4-GlcNAc-Sp5 |
| N023G | Neu5Gc-α-2,6-Gal-β-1,4-GlcNAc-β-1,2-Man-α-1,3-Man-β-1,4-GlcNAc-β-1,4-GlcNAc-Sp5 |
| N025G | Neu5Gc-α-2,3-Gal-β-1,4-(Fuc-α-1,3-)GlcNAc-β-1,2-Man-α-1,3-Man-β-1,4-GlcNAc-β-1,4-GlcNAc-Sp5 |
| N030 | Man-α-1,6-(GlcNAc-β-1,2-Man-α-1,3-)Man-β-1,4-GlcNAc-β-1,4-GlcNAc-Sp5 |
| N210 | GlcNAc-β-1,2-Man-α-1,6-[GlcNAc(3Ac)-β-1,2-Man-α-1,3-Man-β-1,4-GlcNAc-β-1,4-GlcNAc-Sp5 |
| N040 | GlcNAc-β-1,2-Man-α-1,6-Man-β-1,4-GlcNAc-β-1,4-GlcNAc-Sp5 |
| N041 | Gal-β-1,4-GlcNAc-β-1,2-Man-α-1,6-Man-β-1,4-GlcNAc-β-1,4-GlcNAc-Sp5 |
| N042 | Neu5Ac-α-2,3-Gal-β-1,4-GlcNAc-β-1,2-Man-α-1,6-Man-β-1,4-GlcNAc-β-1,4-GlcNAc-Sp5 |
| N043 | Neu5Ac-α-2,6-Gal-β-1,4-GlcNAc-β-1,2-Man-α-1,6-Man-β-1,4-GlcNAc-β-1,4-GlcNAc-Sp5 |
| N044 | Gal-β-1,4-(Fuc-α-1,3-)GlcNAc-β-1,2-Man-α-1,6-Man-β-1,4-GlcNAc-β-1,4-GlcNAc-Sp5 |
| N045 | Neu5Ac-α-2,3-Gal-β-1,4-(Fuc-α-1,3-)GlcNAc-β-1,2-Man-α-1,6-Man-β-1,4-GlcNAc-β-1,4-GlcNAc-Sp5 |
| N050 | GlcNAc-β-1,2-Man-α-1,6-(Man-α-1,3-)Man-β-1,4-GlcNAc-β-1,4-GlcNAc-Sp5 |
| N051 | Gal-β-1,4-GlcNAc-β-1,2-Man-α-1,6-(Man-α-1,3-)Man-β-1,4-GlcNAc-β-1,4-GlcNAc-Sp5 |
| N052 | Neu5Ac-α-2,3-Gal-β-1,4-GlcNAc-β-1,2-Man-α-1,6-(Man-α-1,3-)Man-β-1,4-GlcNAc-β-1,4-GlcNAc-Sp5 |
| N053 | Neu5Ac-α-2,6-Gal-β-1,4-GlcNAc-β-1,2-Man-α-1,6-(Man-α-1,3-)Man-β-1,4-GlcNAc-β-1,4-GlcNAc-Sp |
| N054 | Gal-β-1,4-(Fuc-α-1,3-)GlcNAc-β-1,2-Man-α-1,6-(Man-α-1,3-)Man-β-1,4-GlcNAc-β-1,4-GlcNAc-Sp5 |
| N055 | Neu5Ac-α-2,3-Gal-β-1,4-(Fuc-α-1,3-)GlcNAc-β-1,2-Man-α-1,6-(Man-α-1,3-)Man-β-1,4-GlcNAc-β-1,4-GlcNAc-Sp5 |
| TE001 | Neu5Ac-α-2,6-Gal-β-1,4-GlcNAc-Man-α-1,3-(Neu5Ac-α-2,6-Gal-β-1,4-GlcNAc-Man-α-1,6-)Man-β-1,4-GlcNAc-β-1,4-GlcNAc-β-Asn |
| TE002 | Gal-β-1,4-GlcNAc-β-1,2-Man-α-1,3-(Gal-β-1,4-GlcNAc-β-1,2-Man-α-1,6-)Man-β-1,4-GlcNAc-β-1,4-GlcNAc-β-Asn |
| TE003 | Neu5Gc-α-2,6-Gal-β-1,4-GlcNAc-β-1,2-Man-α-1,3-(Neu5Gc-α-2,6-Gal-β-1,4-GlcNAc-β-1,2-Man-α-1,6-)Man-β-1,4-GlcNAc-β-1,4-GlcNAc-β-Asn |
| TE004 | Neu5Ac-α-2,3-Gal-β-1,4-GlcNAc-β-1,2-Man-α-1,3-(Neu5Ac-α-2,3-Gal-β-1,4-GlcNAc-β-1,2-Man-α-1,6-)Man-β-1,4-GlcNAc-β-1,4-GlcNAc-β-Asn |
| TE005 | Neu5Gc-α-2,3-Gal-β-1,4-GlcNAc-β-1,2-Man-α-1,3-(Neu5Gc-α-2,3-Gal-β-1,4-GlcNAc-β-1,2-Man-α-1,6-)Man-β-1,4-GlcNAc-β-1,4-GlcNAc-β-Asn |
| TE006 | Gal-β-1,4-(Fucα-1,3-)GlcNAc-β-1,2-Man-α-1,3-[Gal-β-1,4-(Fucα-1,3-)GlcNAc-β-1,2-Man-α-1,6-]Man-β-1,4-GlcNAc-β-1,4-GlcNAc-β-Asn |
| TE007 | Gal-α-1,3-Gal-β-1,4-GlcNAc-β-1,2-Man-α-1,3-(Gal-α-1,3-Gal-β-1,4-GlcNAc-β-1,2-Man-α-1,6-)Man-β-1,4-GlcNAc-β-1,4-GlcNAc-β-Asn |
| TE008 | Galα-1,3-Gal-β-1,4-(Fucα-1,3-)GlcNAc-β-1,2-Man-α-1,3-[Galα-1,3-Gal-β-1,4-(Fucα-1,3-)GlcNAc-β-1,2-Man-α-1,6-]Man-β-1,4-GlcNAc-β-1,4-GlcNAc-β-Asn |
| TE009 | Neu5Ac-α-2,8-Neu5Ac-α-2,6-Gal-β-1,4-GlcNAc-β-1,2-Man-α-1,3-(Neu5Ac-α-2,8-Neu5Ac-α-2,6-Gal-β-1,4-GlcNAc-β-1,2-Man-α-1,6-)Man-β-1,4-GlcNAc-β-1,4GlcNAc-β-Asn |
| TE010 | Neu5Gc-α-2,8-Neu5Ac-α-2,6-Gal-β-1,4-GlcNAc-β-1,2-Man-α-1,3-(Neu5Gc-α-2,8-Neu5Ac-α-2,6-Gal-β-1,4-GlcNAc-β-1,2-Man-α-1,6-)Man-β-1,4-GlcNAc-β-1,4GlcNAc-β-Asn |
| TE011 | Neu5Ac-α-2,8-Neu5Gc-α-2,6-Gal-β-1,4-GlcNAc-β-1,2-Man-α-1,3-(Neu5Ac-α-2,8-Neu5Gc-α-2,6-Gal-β-1,4-GlcNAc-β-1,2-Man-α-1,6-)Man-β-1,4-GlcNAc-β-1,4GlcNAc-β-Asn |
| TE012 | Neu5Gc-α-2,8-Neu5Gc-α-2,6-Gal-β-1,4-GlcNAc-β-1,2-Man-α-1,3-(Neu5Gc-α-2,8-Neu5Gc-α-2,6-Gal-β-1,4-GlcNAc-β-1,2-Man-α-1,6-)Man-β-1,4-GlcNAc-β-1,4GlcNAc-β-Asn |
| TE013 | Neu5Ac-α-2,8-Neu5Ac-α-2,3-Gal-β-1,4-GlcNAc-β-1,2-Man-α-1,3-(Neu5Ac-α-2,8-Neu5Ac-α-2,3-Gal-β-1,4-GlcNAc-β-1,2-Man-α-1,6-)Man-β-1,4-GlcNAc-β-1,4GlcNAc-β-Asn |
| TE014 | Neu5Gc-α-2,8-Neu5Ac-α-2,3-Gal-β-1,4-GlcNAc-β-1,2-Man-α-1,3-(Neu5Gc-α-2,8-Neu5Ac-α-2,3-Gal-β-1,4-GlcNAc-β-1,2-Man-α-1,6-)Man-β-1,4-GlcNAc-β-1,4GlcNAc-β-Asn |
| TE015 | Neu5Ac-α-2,8-Neu5Gc-α-2,3-Gal-β-1,4-GlcNAc-β-1,2-Man-α-1,3-(Neu5Ac-α-2,8-Neu5Gc-α-2,3-Gal-β-1,4-GlcNAc-β-1,2-Man-α-1,6-)Man-β-1,4-GlcNAc-β-1,4GlcNAc-β-Asn |
| TE016 | Neu5Gc-α-2,8-Neu5Gc-α-2,3-Gal-β-1,4-GlcNAc-β-1,2-Man-α-1,3-(Neu5Gc-α-2,8-Neu5Gc-α-2,3-Gal-β-1,4-GlcNAc-β-1,2-Man-α-1,6-)Man-β-1,4-GlcNAc-β-1,4GlcNAc-β-Asn |
| TE017 | Neu5Ac-α-2,3-Gal-β-1,4-(Fucα-1,3-)GlcNAc-β-1,2-Man-α-1,3-[Neu5Ac-α-2,3-Gal-β-1,4-(Fucα-1,3-)GlcNAc-β-1,2-Man-α-1,6-]Man-β-1,4-GlcNAc-β-1,4-GlcNAc-βAsn |
| TE018 | Neu5Gc-α-2,3-Gal-β-1,4-(Fucα-1,3-)GlcNAc-β-1,2-Man-α-1,3-[Neu5Gc-α-2,3-Gal-β-1,4-(Fucα-1,3-)GlcNAc-β-1,2-Man-α-1,6-]Man-β-1,4-GlcNAc-β-1,4-GlcNAc-βAsn |
| TE019 | Neu5Ac-α-2,3-Gal-β-1,4-GlcNAc-β-1,3-Gal-β-1,4-GlcNAc-β-1,2-Man-α-1,3-(Neu5Ac-α-2,3-Gal-β-1,4-GlcNAc-β-1,3-Gal-β-1,4-GlcNAc-β-1,2-Man-α-1,6-)Man-β1,4-GlcNAc-β-1,4-GlcNAc-β-Asn |
| TE020 | Neu5Gc-α-2,3-Gal-β-1,4-GlcNAc-β-1,3-Gal-β-1,4-GlcNAc-β-1,2-Man-α-1,3-(Neu5Gc-α-2,3-Gal-β-1,4-GlcNAc-β-1,3-Gal-β-1,4-GlcNAc-β-1,2-Man-α-1,6-)Man-β1,4-GlcNAc-β-1,4-GlcNAc-β-Asn |
| TE021 | Neu5Ac-α-2,6-Gal-β-1,4-GlcNAc-β-1,3-(Neu5AC-α-2,6-)Gal-β-1,4-GlcNAc-β-1,2-Man-α-1,3-[Neu5Ac-α-2,6-Gal-β-1,4-GlcNAc-β-1,3-(Neu5AC-α-2,6-)Gal-β-1,4GlcNAc-β-1,2-Man-α-1,6-]Man-β-1,4-GlcNAc-β-1,4-GlcNAc-β-Asn |
| TE022 | Neu5Gc-α-2,6-Gal-β-1,4-GlcNAc-β-1,3-(Neu5GC-α-2,6-)Gal-β-1,4-GlcNAc-β-1,2-Man-α-1,3-[Neu5Gc-α-2,6-Gal-β-1,4-GlcNAc-β-1,3-(Neu5GC-α-2,6-)Gal-β-1,4GlcNAc-β-1,2-Man-α-1,6-]Man-β-1,4-GlcNAc-β-1,4-GlcNAc-β-Asn |
| TE023 | Neu5Ac-α-2,3-Gal-β-1,4-GlcNAc-β-1,3-(Neu5AC-α-2,6-)Gal-β-1,4-GlcNAc-β-1,2-Man-α-1,3-[Neu5Ac-α-2,3-Gal-β-1,4-GlcNAc-β-1,3-(Neu5AC-α-2,6-)Gal-β-1,4GlcNAc-β-1,2-Man-α-1,6-]Man-β-1,4-GlcNAc-β-1,4-GlcNAc-β-Asn |
| TE024 | GlcNAc-β-1,3-Gal-β-1,4-GlcNAc-β-1,2-Man-α-1,3-(GlcNAc-β-1,3-Gal-β-1,4-GlcNAc-β-1,2-Man-α-1,6-)Man-β-1,4-GlcNAc-β-1,4-GlcNAc-β-Asn |
| TE025 | Gal-β-1,4-GlcNAc-β-1,3-Gal-β-1,4-GlcNAc-β-1,2-Man-α-1,3-(Gal-β-1,4-GlcNAc-β-1,3-Gal-β-1,4-GlcNAc-β-1,2-Man-α-1,6-)Man-β-1,4-GlcNAc-β-1,4-GlcNAc-β-Asn |
| TE026 | GlcNAc-β-1,3-Gal-β-1,4-GlcNAc-β-1,3-Gal-β-1,4-GlcNAc-β-1,2-Man-α-1,3-(GlcNAc-β-1,3-Gal-β-1,4-GlcNAc-β-1,3-Gal-β-1,4-GlcNAc-β-1,2-Man-α-1,6-)Man-β-1,4GlcNAc-β-1,4-GlcNAc-β-Asn |
| TE027 | Gal-β-1,4-GlcNAc-β-1,3-Gal-β-1,4-GlcNAc-β-1,3-Gal-β-1,4-GlcNAc-β-1,2-Man-α-1,3-(Gal-β-1,4-GlcNAc-β-1,3-Gal-β-1,4-GlcNAc-β-1,3-Gal-β-1,4-GlcNAc-β-1,2Man-α-1,6-)Man-β-1,4-GlcNAc-β-1,4-GlcNAc-β-Asn |
| TE028 | GlcNAc-β-1,3-Gal-β-1,4-GlcNAc-β-1,3-Gal-β-1,4-GlcNAc-β-1,3-Gal-β-1,4-GlcNAc-β-1,2-Man-α-1,3-(GlcNAc-β-1,3-Gal-β-1,4-GlcNAc-β-1,3-Gal-β-1,4-GlcNAc-β1,3-Gal-β-1,4-GlcNAc-β-1,2-Man-α-1,6-)Man-β-1,4-GlcNAc-β-1,4-GlcNAc-β-Asn |
| TE029 | Gal-β-1,4-GlcNAc-β-1,3-Gal-β-1,4-GlcNAc-β-1,3-Gal-β-1,4-GlcNAc-β-1,3-Gal-β-1,4-GlcNAc-β-1,2-Man-α-1,3-(Gal-β-1,4-GlcNAc-β-1,3-Gal-β-1,4-GlcNAc-β-1,3Gal-β-1,4-GlcNAc-β-1,3-Gal-β-1,4-GlcNAc-β-1,2-Man-α-1,6-)Man-β-1,4-GlcNAc-β-1,4-GlcNAc-β-Asn |
| TE030 | GlcNAc-β-1,3-Gal-β-1,4-GlcNAc-β-1,3-Gal-β-1,4-GlcNAc-β-1,3-Gal-β-1,4-GlcNAc-β-1,3-Gal-β-1,4-GlcNAc-β-1,2-Man-α-1,3-(GlcNAc-β-1,3-Gal-β-1,4-GlcNAc-β1,3-Gal-β-1,4-GlcNAc-β-1,3-Gal-β-1,4-GlcNAc-β-1,3-Gal-β-1,4-GlcNAc-β-1,2-Man-α-1,6-)Man-β-1,4-GlcNAc-β-1,4-GlcNAc-β-Asn |
| TE031 | Gal-β-1,4-GlcNAc-β-1,3-Gal-β-1,4-GlcNAc-β-1,3-Gal-β-1,4-GlcNAc-β-1,3-Gal-β-1,4-GlcNAc-β-1,3-Gal-β-1,4-GlcNAc-β-1,2-Man-α-1,3-(Gal-β-1,4-GlcNAc-β-1,3Gal-β-1,4-GlcNAc-β-1,3-Gal-β-1,4-GlcNAc-β-1,3-Gal-β-1,4-GlcNAc-β-1,3-Gal-β-1,4-GlcNAc-β-1,2-Man-α-1,6-)Man-β-1,4-GlcNAc-β-1,4-GlcNAc-β-Asn |
| TE032 | GlcNAc-β-1,3-Gal-β-1,4-(Fucα-1,3-)GlcNAc-β-1,2-Man-α-1,3-[GlcNAc-β-1,3-Gal-β-1,4-(Fucα-1,3-)GlcNAc-β-1,2-Man-α-1,6-]Man-β-1,4-GlcNAc-β-1,4-GlcNAc-βAsn |
| TE033 | Gal-β-1,4-GlcNAc-β-1,3-Gal-β-1,4-(Fucα-1,3-)GlcNAc-β-1,2-Man-α-1,3-[Gal-β-1,4-GlcNAc-β-1,3-Gal-β-1,4-(Fucα-1,3-)GlcNAc-β-1,2-Man-α-1,6-]Man-β-1,4GlcNAc-β-1,4-GlcNAc-β-Asn |
| TE034 | Neu5Ac-α-2,3-Gal-β-1,4-GlcNAc-β-1,3-Gal-β-1,4-(Fucα-1,3-)GlcNAc-β-1,2-Man-α-1,3-[Neu5Ac-α-2,3-Gal-β-1,4-GlcNAc-β-1,3-Gal-β-1,4-(Fucα-1,3-)GlcNAc-β-1,2Man-α-1,6-]Man-β-1,4-GlcNAc-β-1,4-GlcNAc-β-Asn |
| TE035 | GlcNAc-β-1,3-Gal-β-1,4-GlcNAc-β-1,3-Gal-β-1,4-(Fucα-1,3-)GlcNAc-β-1,2-Man-α-1,3-[GlcNAc-β-1,3-Gal-β-1,4-GlcNAc-β-1,3-Gal-β-1,4-(Fucα-1,3-)GlcNAc-β-1,2Man-α-1,6-]Man-β-1,4-GlcNAc-β-1,4-GlcNAc-β-Asn |
| TE036 | Gal-β-1,4-GlcNAc-β-1,3-Gal-β-1,4-GlcNAc-β-1,3-Gal-β-1,4-(Fucα-1,3-)GlcNAc-β-1,2-Man-α-1,3-[Gal-β-1,4-GlcNAc-β-1,3-Gal-β-1,4-GlcNAc-β-1,3-Gal-β-1,4-(Fucα1,3-)GlcNAc-β-1,2-Man-α-1,6-]Man-β-1,4-GlcNAc-β-1,4-GlcNAc-β-Asn |
| TE037 | GlcNAc-β-1,3-Gal-β-1,4-GlcNAc-β-1,3-Gal-β-1,4-GlcNAc-β-1,3-Gal-β-1,4-(Fucα-1,3-)GlcNAc-β-1,2-Man-α-1,3-[GlcNAc-β-1,3-Gal-β-1,4-GlcNAc-β-1,3-Gal-β-1,4GlcNAc-β-1,3-Gal-β-1,4-(Fucα-1,3-)GlcNAc-β-1,2-Man-α-1,6-]Man-β-1,4-GlcNAc-β-1,4-GlcNAc-β-Asn |
